# Stable reliability diagrams for probabilistic classifiers

**DOI:** 10.1073/pnas.2016191118

**Published:** 2021-02-17

**Authors:** Timo Dimitriadis, Tilmann Gneiting, Alexander I. Jordan

**Affiliations:** ^a^Alfred Weber Institute of Economics, Heidelberg University, 69115 Heidelberg, Germany;; ^b^Computational Statistics Group, Heidelberg Institute for Theoretical Studies, 69118 Heidelberg, Germany;; ^c^Institute for Stochastics, Karlsruhe Institute of Technology, 76131 Karlsruhe, Germany

**Keywords:** calibration, discrimination ability, probability forecast, score decomposition, weather prediction

## Abstract

A probability forecast or probabilistic classifier is reliable or calibrated if the predicted probabilities are matched by ex post observed frequencies, as examined visually in reliability diagrams. The classical binning and counting approach to plotting reliability diagrams has been hampered by a lack of stability under unavoidable, ad hoc implementation decisions. Here, we introduce the CORP approach, which generates provably statistically consistent, optimally binned, and reproducible reliability diagrams in an automated way. CORP is based on nonparametric isotonic regression and implemented via the pool-adjacent-violators (PAV) algorithm—essentially, the CORP reliability diagram shows the graph of the PAV-(re)calibrated forecast probabilities. The CORP approach allows for uncertainty quantification via either resampling techniques or asymptotic theory, furnishes a numerical measure of miscalibration, and provides a CORP-based Brier-score decomposition that generalizes to any proper scoring rule. We anticipate that judicious uses of the PAV algorithm yield improved tools for diagnostics and inference for a very wide range of statistical and machine learning methods.

## Significance

Probabilistic classifiers assign predictive probabilities to binary events, such as rainfall tomorrow, a recession, or a personal health outcome. Such a system is reliable or calibrated if the predictive probabilities are matched by the observed frequencies. In practice, calibration is assessed graphically in reliability diagrams and quantified via the reliability component of mean scores. Extant approaches rely on binning and counting and have been hampered by ad hoc implementation decisions, a lack of reproducibility, and inefficiency. Here, we introduce the CORP approach, which uses the pool-adjacent-violators algorithm to generate optimally binned, reproducible, and provably statistically consistent reliability diagrams, along with a numerical measure of miscalibration based on a revisited score decomposition.

Calibration or reliability is a key requirement on any probability forecast or probabilistic classifier. In a nutshell, a probabilistic classifier assigns a predictive probability to a binary event. The classifier is calibrated or reliable if, when looking back at a series of extant forecasts, the conditional event frequencies match the predictive probabilities. For example, if we consider all cases with a predictive probability of about 0.80, the observed event frequency ought to be about 0.80 as well. While for many decades, researchers and practitioners have been checking calibration in myriads of applications (1, 2), the topic is subject to a surge of interest in machine learning (3), spurred by the recent recognition that “modern neural networks are uncalibrated, unlike those from a decade ago” (4).

## Reliability Diagrams: Binning and Counting

The key diagnostic tool for checking calibration is the reliability diagram, which plots the observed event frequency against the predictive probability. In discrete settings, where there are only a few predictive probabilities, such as, e.g., $0,\frac{1}{10},\ldots ,\frac{9}{10},1$, this is straightforward. However, even in discrete settings, there might be many such values. Furthermore, statistical and machine-learning approaches to binary classification generate continuous predictive probabilities that can take any value between zero and one, and typically the forecast values are pairwise distinct. In these settings, researchers have been using the “binning and counting” approach, which starts by selecting a certain, typically arbitrary, number of bins for the forecast values. Then, for each bin, one plots the respective conditional event frequency versus the midpoint or average forecast value in the bin. For calibrated or reliable forecasts, the two quantities ought to match, and so the points plotted ought to lie on, or close to, the diagonal (2, 5).

**Fig. 1.**
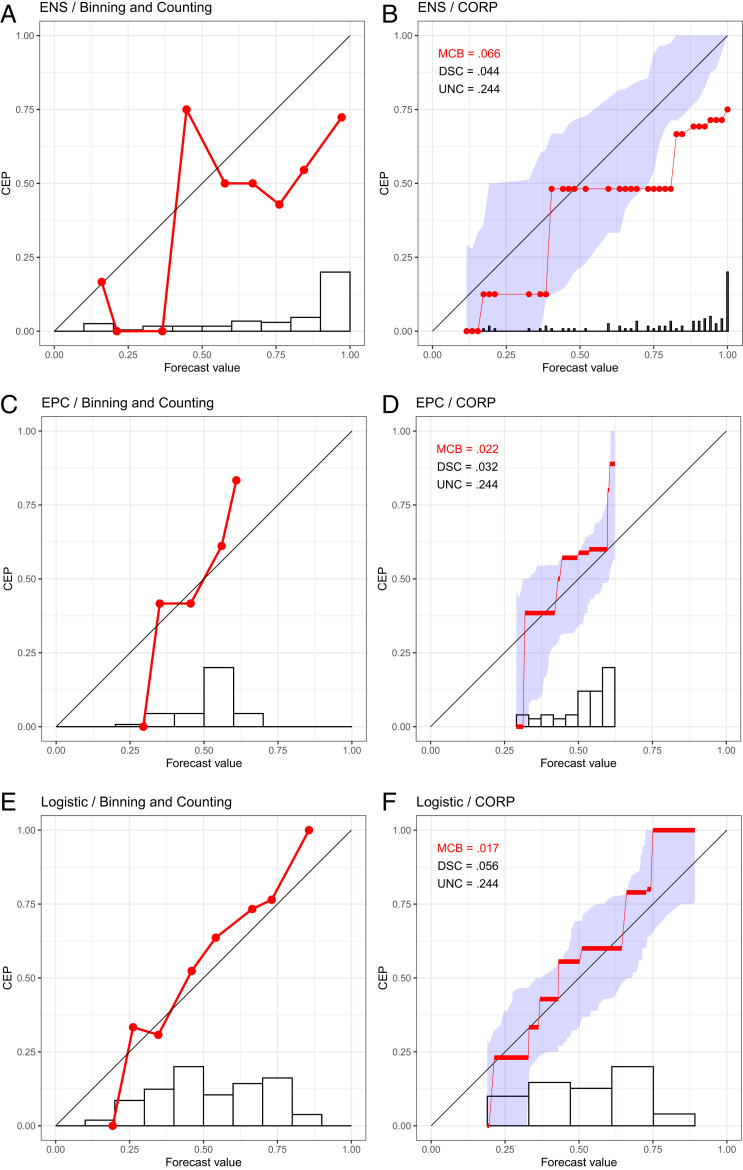
Reliability diagrams for probability of precipitation forecasts over Niamey, Niger (7), in July–September 2016 under ENS (*A* and *B*), EPC (*C* and *D*), and Logistic (*E* and *F*) methods. (*A*, *C*, and *E*) We show reliability diagrams under the binning and counting approach with a choice of 10 equally spaced bins. (*B*, *D*, and *F*) We show CORP reliability diagrams with uncertainty quantification through 90% consistency bands. The histograms at the bottom illustrate the distribution of the n=92 forecast values.

**Fig. 2.**
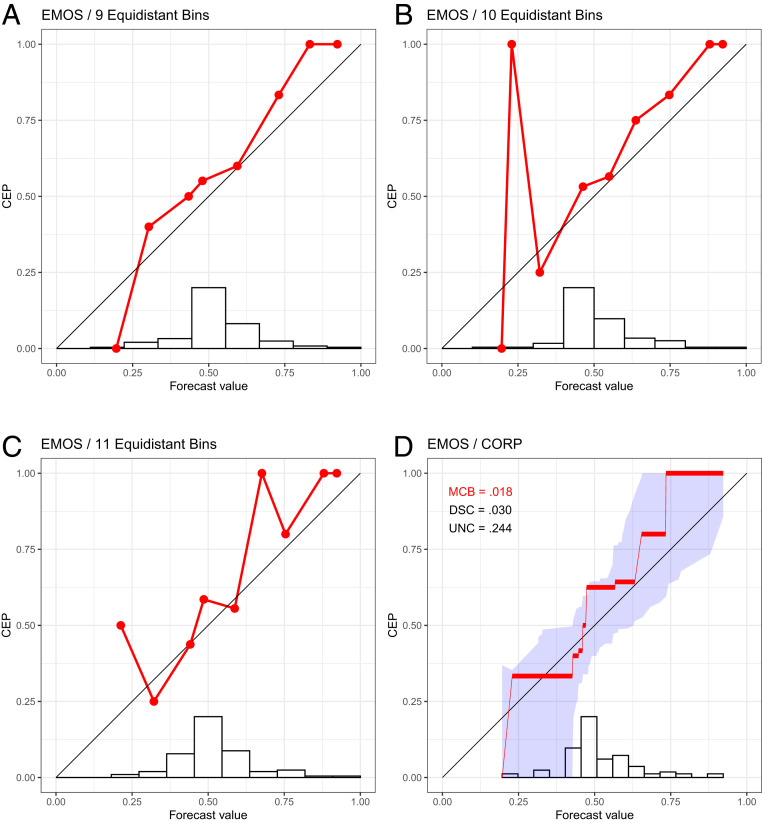
Reliability diagrams for probability of precipitation forecasts over Niamey, Niger (7), in July–September 2016 with the EMOS method, using the binning and counting approach with a choice of 9 (*A*), 10 (*B*), and 11 (*C*) equidistant bins, together with the CORP reliability diagram (*D*), for which we provide uncertainty quantification through 90% consistency bands.

In Fig. 1 *A*, *C* and *E*, we show reliability diagrams based on the binning and counting approach with a choice of m=10 equally spaced bins for 24-h-ahead daily probability of precipitation forecasts at Niamey, Niger, in July–September 2016. They concern three competing forecasting methods, including the world-leading, 52-member ensemble system run by the European Center for Medium-Range Weather Forecasts [ENS (6)], a reference forecast called extended probabilistic climatology (EPC), and a purely data-driven statistical forecast (Logistic), as described by Vogel et al. (ref. 7, figure 2).

Not surprisingly, the classical approach to plotting reliability diagrams is highly sensitive to the specification of the bins, and the visual appearance may change drastically under the slightest change. We show an example in Fig. 2 *A*–*C* for a fourth type of forecast at Niamey, namely, a statistically postprocessed version of the ENS forecast called ensemble model output statistics (EMOS), for which choices of m=9, 10, or 11 equidistant bins yield drastically distinct reliability diagrams. This is a disconcerting state of affairs for a widely used data-analytic tool and contrary to well-argued recent pleas for reproducibility (8) and stability (9).

A simple and seemingly effective enhancement is to use evenly populated bins, as opposed to equidistantly spaced bins. Perhaps surprisingly, instability remains a major issue, typically caused by multiple occurrences of the same forecast value at bin breaks. Furthermore, the instabilities carry over to associated numerical measures of calibration, such as the Brier-score reliability component (10–14) and the Hosmer–Lemeshow statistic (15–19). These issues have been well documented in both research papers (16–20) and textbooks (21–23) and may occur even when the size n of the dataset is large. See *SI Appendix*, sections S1 and S2 for illustrations on meteorological, geophysical, social science, and economic forecast datasets.

While alternative methods for the choice of the binning have been proposed in the literature (5, 24, 25), extant approaches exhibit similar instabilities, lack theoretical justification, are elaborate, and have not been adopted by practitioners. Instead, researchers across disciplines continue to craft reliability diagrams and report associated measures of calibration based on ad hoc choices. In this light, Stephenson et al. (ref. 26, p. 757) call for the development of “nonparametric approaches for estimating the reliability curves (and hence the Brier score components), which also include[d] point-wise confidence intervals.”

Here, we introduce an approach to reliability diagrams and score decompositions, which resolves these issues in a theoretically optimal and readily implementable way, as illustrated on the forecasts at Niamey in Figs. 1 *B*, *D*, and *F* and 2*D*. In a nutshell, we use nonparametric isotonic regression and the pool-adjacent-violators (PAV) algorithm to estimate conditional event probabilities (CEPs), which yields a fully automated choice of bins that adapts to both discrete and continuous settings, without any need for tuning parameters or implementation decisions. We equip the diagram with quantitative measures of (mis)calibration (MCB), discrimination ability (DSC), and uncertainty (UNC), which improve upon the classical Brier-score decomposition in terms of stability. We call this stable approach CORP, as its novelty and power include the following four properties.

### Consistency.

The CORP reliability diagram and the MCB measure of (mis)calibration are consistent in the classical statistical sense of convergence to population characteristics. We leverage existing asymptotic theory (27–29) to demonstrate that the rate of convergence is best possible and to generate large sample consistency and confidence bands for uncertainty quantification.

### Optimality.

The CORP reliability diagram is optimally binned, in that no other choice of bins generates more skillful (re)calibrated forecasts, subject to regularization via isotonicity (ref. 30, theorem 1.10, and refs. 31 and 32).

### Reproducibility.

The CORP approach does not require any tuning parameters or implementation decision, thus yielding well-defined and readily reproducible reliability diagrams and score decompositions.

### PAV Algorithm-Based.

CORP is based on nonparametric isotonic regression and implemented via the PAV algorithm, a classical iterative procedure with linear complexity only (33, 34). Essentially, the CORP reliability diagram shows the graph of the PAV-(re)calibrated forecast probabilities.

In the remainder of the article, we provide the details of CORP reliability diagrams and score decompositions, and we substantiate the above claims via mathematical analysis and simulation experiments.

## The CORP Approach: Optimal Binning via the PAV Algorithm

The basic idea of CORP is to use nonparametric isotonic regression to estimate a forecast’s CEPs as a monotonic, nondecreasing function of the original forecast values. Fortunately, in this simple setting, there is one, and only one, kind of nonparametric isotonic regression, for which the PAV algorithm provides a simple algorithmic solution (33, 34). To each original forecast value, the PAV algorithm assigns a (re)calibrated probability under the regularizing constraint of isotonicity, as illustrated in textbooks (ref. 35, figures 2.13 and 10.7), and this solution is optimal under a very broad class of loss functions (ref. 30, theorem 1.10). In particular, the PAV solution constitutes both the nonparametric isotonic least squares and the nonparametric isotonic maximum-likelihood estimate of the CEPs.

The CORP reliability diagram plots the PAV-calibrated probability versus the original forecast value, as illustrated on the Niamey data in Figs. 1 *B*, *D*, and *F* and 2*D*. The PAV algorithm assigns calibrated probabilities to the individual unique forecast values, and we interpolate linearly in between, to facilitate comparison with the diagonal that corresponds to perfect calibration. If a group of (one or more) forecast values are assigned identical PAV-calibrated probabilities, the CORP reliability diagram displays a horizontal segment. The horizontal sections can be interpreted as bins, and the respective PAV-calibrated probabilities are simply the bin-specific empirical event frequencies. For example, we see from Fig. 1*B* that the PAV algorithm assigns a calibrated probability of 0.125 to ENS forecast values between $\frac{9}{52}$ and $\frac{20}{52}$ and a calibrated probability of 0.481 to ENS values between $\frac{21}{52}$ and $\frac{42}{52}$. The PAV algorithm guarantees that both the number and the positions of the horizontal segments (and, hence, the bins) in the CORP reliability diagram are determined in a fully automated, optimal way.

The assumption of nondecreasing CEPs is natural, as decreasing estimates are counterintuitive, routinely being dismissed as artifacts by practitioners. Furthermore, the constraint provides an implicit regularization, serving to stabilize the estimate and counteract overfitting, despite the method being entirely nonparametric. Under the binning and counting approach, small or sparsely populated bins are subject to overfitting and large estimation uncertainty, as exemplified by the sharp upward spike in Fig. 2*B*. The assumption of isotonicity in CORP stabilizes the estimate and avoids artifacts; see the examples in Fig. 2*D* and *SI Appendix*, Figs. S2–S5.

In contrast to the binning and counting approach, which has not been subject to asymptotic analysis, CORP reliability diagrams are provably statistically consistent: If the predictive probabilities and event realizations are samples from a fixed, joint distribution, then the graph of the diagram converges to the respective population equivalent, as a direct consequence of existing large sample theory for nonparametric isotonic regression estimates (27–29). Furthermore, CORP is asymptotically efficient, in the sense that its automated choice of binning results in an estimate that is as accurate as possible in the large sample limit. In *Appendix B*, we formalize these arguments and report on a simulation study, for which we give details in *Appendix A*, and which demonstrates that the efficiency of the CORP approach also holds in small samples.

**Fig. 3.**
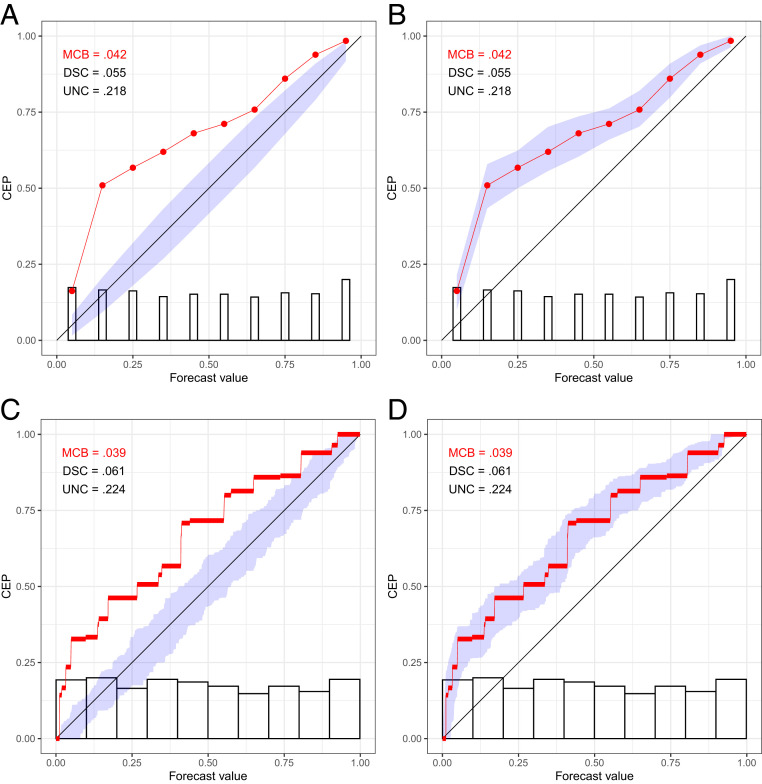
CORP reliability diagrams in the setting of discretely (*A* and *B*) and continuously (*C* and *D*), uniformly distributed, simulated predictive probabilities x with a true, miscalibrated CEP of $\sqrt{x}$, with uncertainty quantification via consistency (*A* and *C*) and confidence (*B* and *D*) bands at the 90% level.

Traditionally, reliability diagrams have been accompanied by histograms or bar plots of the marginal distribution of the predictive probabilities, on either standard or logarithmic scales (e.g., ref. 36). Under the binning and counting approach, the histogram bins are typically the same as the reliability bins. In plotting CORP reliability diagrams, we distinguish discretely and continuously distributed classifiers or forecasts. Intuitively, the discrete case refers to forecast values that only take on a finite and sufficiently small number of distinct values. Then, we show the PAV-calibrated probabilities as dots, interpolate linearly in between, and visualize the marginal distribution of the forecast values in a bar diagram, as illustrated in Fig. 3 *A* and *B*. For continuously distributed forecasts, essentially every forecast takes on a different value, whence the choice of binning becomes crucial. The CORP reliability diagram displays the bin-wise constant PAV-calibrated probabilities in horizontal segments, which are linearly interpolated in between, and we use the Freedman–Diaconis rule (37) to generate a histogram estimate of the marginal density of the forecast values, as exemplified in Fig. 3 *C* and *D*. In our software implementation (38), a simple default is used: If the smallest distance between any two distinct forecast values is 0.01 or larger, we operate in the discrete setting, and else in the continuous one. The CORP reliability diagrams in Figs. 1–3 also display measures of (most importantly, and hence highlighted) (mis)calibration (MCB), discrimination (DSC), and uncertainty (UNC), discussed in detail later on as we introduce the CORP score decomposition.

## Uncertainty Quantification

Bröcker and Smith (39) convincingly advocate the need for uncertainty quantification, so that structural deviations of the estimated CEP from the diagonal can be distinguished from deviations that merely reflect noise. They employ a resampling technique for the binning and counting method in order to find consistency bands under the assumption of calibration. For CORP, we extend this approach in two crucial ways, by generating either consistency or confidence bands and by using either a resampling technique or asymptotic distribution theory, where we leverage existing theory for nonparametric isotonic regression estimates (27–29).

Consistency bands are generated under the assumption that the probability forecasts are calibrated, and so they are positioned around the diagonal. There is a close relation to the classical interpretation of statistical tests and *P* values: Under the hypothesized perfect calibration, how much do reliability diagrams vary, and how (un)likely is the outcome at hand? In contrast, confidence bands cluster around the CORP estimate and follow the classical interpretation of frequentist confidence intervals: If one repeats the experiment numerous times, the fraction of confidence intervals that contain the true CEP approaches the nominal level. The two methods are illustrated in Fig. 3, where the diagrams in Fig. 3 *B* and *D* feature confidence bands and in Fig. 3 *A* and *C* show consistency bands, as do the CORP reliability diagrams in Figs. 1 *B*, *D*, and *F* and 2*D*.

In our adaptation of the resampling approach, for each iteration, the resampled CORP reliability diagram is computed, and confidence or consistency bands are then specified by using resampling percentiles, in customary ways. For consistency bands, the resampling is based on the assumption of calibrated original forecast values, whereas PAV-calibrated probabilities are used to generate confidence bands. While resampling works well in small to medium samples, the use of asymptotic theory suits cases where the sample size n of the dataset is large—exactly when the computational cost of resampling-based procedures becomes prohibitive. Existing asymptotic theory is readily applicable and operates under weak conditions on the marginal distribution of the forecast values and (strict) monotonicity and smoothness of (true) CEPs (27–29).

The distinction between discretely and continuously distributed forecasts becomes critical here, as the asymptotic theory differs between these cases. For discrete forecasts, results of El Barmi and Mukerjee (27) imply that the difference between the estimated and the true CEP, scaled by $n^{1/2}$, converges to a (mixture of) normal distribution(s). For continuous forecasts, following Wright (28), the difference between the estimated and the true CEP, magnified by $n^{1/3}$, converges to Chernoff’s distribution (40). The distinct scaling laws imply that the convergence is faster in the discrete than in the continuous case, since in the former, the CORP binning stabilizes as it captures the discrete forecast values, and, thereafter, the amount of samples per bin increases linearly, in accordance with the standard $n^{1/2}$ rate. In either setting, asymptotic consistency and confidence bands can be obtained from quantiles of the asymptotic distributions in customary ways. See *SI Appendix*, section S3 for details on both the resampling algorithm and asymptotic theory. As a caveat, these techniques operate under the assumption of independent, or at least exchangeable, forecast cases, which may or may not be warranted in practice. We encourage follow-up work in dependent data settings, as recently tackled for related types of data-science tools (41).

**Fig. 4.**
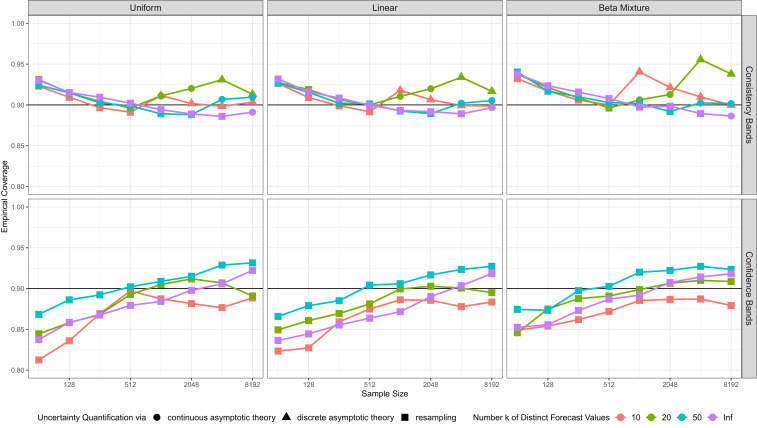
Empirical coverage, averaged equally over the forecast values, of 90% uncertainty bands for CORP reliability diagrams under default choices for 1,000 simulation replicates. *Upper* concerns consistency bands, and *Lower* confidence bands. The columns correspond to three types of marginal distributions for the forecast values, and colors distinguish discrete and continuous settings, as described in *Appendix A*. Different symbols denote reliance of the bands on resampling, discrete, or continuous asymptotic distribution theory.

In our software implementation (38), we use the following default choices. Suppose that the sample size is n, and there are k unique forecast values. For consistency bands, if *n* ≤ 1,000 or if *n* ≤ 5,000 and n≤50k, we use resampling; else we rely on asymptotic theory. In the latter case, we employ the discrete asymptotic distribution if $n\geq 8k^{2}$, while otherwise we use the continuous one. For confidence bands, the current default uses resampling throughout, as the asymptotic theory depends on the assumption of a true CEP with strictly positive derivative. In the simulation examples in Fig. 3, which are based on *n* = 1,024 observations, this implies the use of resampling in Fig. 3 *B*–*D* and of discrete asymptotic theory in Fig. 3*A*. Fig. 4 shows coverage rates of 90% consistency and confidence bands in the simulation settings described in *Appendix A*, based on the default choices. The coverage rates are generally accurate, or slightly conservative, especially in large samples. In *SI Appendix*, section S4*A*, we qualitatively confirm these results in simulation settings driven by datasets from meteorology, astrophysics, social science, and economics.

## CORP Score Decomposition: MCB, DSC, and UNC Components

Scoring rules provide a numerical measure of the quality of a classifier or forecast by assigning a score or penalty S(x,y), based on forecast value x∈[0,1] for a dichotomous event y∈{0,1}. A scoring rule is proper (42) if it assigns the minimal penalty in expectation when x equals the true underlying event probability. If the minimum is unique, the scoring rule is strictly proper. In practice, for a given sample $\left(x_{1},y_{1}\right),\ldots ,\left(x_{n},y_{n}\right)$ of forecast-realization pairs, the empirical score

$${\bar{\text{S}}}_{\text{X}}=\frac{1}{n}\overset{n}{\underset{i=1}{\sum }}\text{S}\left(x_{i},y_{i}\right),$$ [1]

is used for forecast ranking. Table 1 presents examples of proper and strictly proper scoring rules. The Brier score and logarithmic score are strictly proper. In contrast, the misclassification error is proper, but not strictly proper—all that matters is whether or not a classifier probability is on the correct side of $\frac{1}{2}$.

**Table 1.** Scoring rules for probability forecasts of binary events

Score	Propriety	Analytic form of S(x,y)
Brier	Strict	${\left(x-y\right)}^{2}$
Logarithmic	Strict	−y⁡log⁡x−(1−y)log(1−x)
Misclassification error	Nonstrict	$1\left(x<\frac{1}{2},y=1\right)+1\left(x>\frac{1}{2},y=0\right)+\frac{1}{2}\;1\left(x=\frac{1}{2}\right)$

Under any proper scoring rule, the mean score ${\bar{\text{S}}}_{\text{X}}$ constitutes a measure of overall predictive performance. For several decades, researchers have been seeking to decompose ${\bar{\text{S}}}_{\text{X}}$ into intuitively appealing components, typically thought of as reliability (REL), resolution (RES), and uncertainty (UNC) terms. The REL component measures how much the conditional event frequencies deviate from the forecast probabilities, while RES quantifies the ability of the forecasts to discriminate between events and nonevents. Finally, UNC measures the inherent difficulty of the prediction problem, but does not depend on the forecast under consideration. While there is a consensus on the character and intuitive interpretation of the decomposition terms, their exact form remains subject to debate, despite a half-century quest in the wake of Murphy’s (11) Brier-score decomposition. In particular, Murphy’s decomposition is exact in the discrete case, but fails to be exact under continuous forecasts, which has prompted the development of increasingly complex types of decompositions (13, 26).

Here, we adopt the general score decomposition introduced by Dawid (12), advocated forcefully by Siegert (14), and discussed by various other authors as well (e.g., refs. 13 and 43). Specifically, let ${\bar{\text{S}}}_{\text{X}}$,

$${\bar{\text{S}}}_{\text{C}}=\frac{1}{n}\overset{n}{\underset{i=1}{\sum }}\text{S}\left({\hat{x}}_{i},y_{i}\right),\;and\;{\bar{\text{S}}}_{\text{R}}=\frac{1}{n}\overset{n}{\underset{i=1}{\sum }}\text{S}\left(r,y_{i}\right)$$ [2]

denote the mean score for the original forecast values of Eq. **1**, the mean score for suitably (re)calibrated probabilities ${\hat{x}}_{1},\ldots ,{\hat{x}}_{n}$, and the mean score for a constant reference forecast r, respectively. Then, ${\bar{\text{S}}}_{\text{X}}$ decomposes as

$${\bar{\text{S}}}_{\text{X}}=\underset{\text{MCB}}{\underset{︸}{\left({\bar{\text{S}}}_{\text{X}}-{\bar{\text{S}}}_{\text{C}}\right)}}-\underset{\text{DSC}}{\underset{︸}{\left({\bar{\text{S}}}_{\text{R}}-{\bar{\text{S}}}_{\text{C}}\right)}}+\underset{\text{UNC}}{\underset{︸}{{\bar{\text{S}}}_{\text{R}}}},$$ [3]

where we adopt, in part, terminology proposed by Ehm and Ovcharov (44) and Pohle (45). As defined in Eq. **3**, the miscalibration component MCB is the difference of the mean scores of the original and the (re)calibrated forecasts. Similarly, the DSC component quantifies discrimination ability via the difference between the mean score for the reference and the (re)calibrated forecast, while the classical measure of uncertainty (UNC) is simply the mean score for the reference forecast.

In the extant literature, it has been assumed implicitly or explicitly that the (re)calibrated and reference forecasts can be chosen at researchers’ discretion (e.g., refs. 14 and 45), without considering whether or not the transformed probabilities are calibrated in the classical technical sense. Specifically, a probability forecast with unique forecast values $z_{1}<\cdots <z_{k}$ that are issued $n_{1},\ldots ,n_{k}$ times, with $o_{1},\ldots ,o_{k}$ of these cases being events, is “calibrated” if

$$z_{j}=\frac{o_{j}}{n_{j}}\;\text{for all}\;j=1,\ldots ,k.$$ [4]

We posit that in the score decomposition of Eq. **3** the (re)calibrated values ${\hat{x}}_{1},\ldots {\hat{x}}_{n}$ ought to be the PAV-transformed probabilities, as displayed in the CORP reliability diagram, whereas the reference forecast r ought to be the marginal event frequency $ȳ=\frac{1}{n}\sum_{i=1}^{n}y_{i}$. These forecasts both satisfy the calibration condition of Eq. **4**.

We refer to the resulting decomposition as the CORP score decomposition, which enjoys the following properties:

∙ MCB≥0 with equality if the original forecast is calibrated.
∙ DSC≥0 with equality if the PAV-(re)calibrated forecast is constant.
∙ The decomposition is exact.

In particular, the CORP score decomposition never yields counterintuitive negative values of the components, contrary to choices in the extant literature. The cases of vanishing components (MCB=0 or DSC=0) support the intuitive interpretation of CORP reliability diagrams, in that parts away from the diagonal indicate lack of calibration, whereas extended horizontal segments are indicative of diminished discrimination ability. For refined technical statements, proofs, and a demonstration that under (re)calibration methods other than isotonic regression these properties may fail, see *Appendix C* and *SI Appendix*, section S5.

If S is the Brier score, then in the special case of discrete forecasts with nondecreasing CEPs, the MCB, DSC, and UNC terms in Eq. **3** agree with the REL, RES, and UNC components, respectively, in the classical Murphy decomposition, as we demonstrate in Theorem 2 in *Appendix C*. If S is the misclassification error, MCB equals the fraction of cases in which the PAV-calibrated probability was on the correct side of $\frac{1}{2}$, but the original forecast value was not, minus the fraction vice versa, with natural adaptations in the case of ties.

In Table 2, we illustrate the CORP Brier-score decomposition for the probability of precipitation forecasts at Niamey in Figs. 1 and 2. The purely data-driven Logistic forecast obtains the best (smallest) mean score, the best (smallest) MCB term, and the best (highest) DSC component, well in line with the insights offered by the CORP reliability diagrams and attesting to the particular challenges for precipitation forecasts over northern tropical Africa (7).

**Table 2.** CORP Brier-score decomposition for the probability of precipitation forecasts in Figs. 1, 2, and 5

Forecast	${\bar{\text{S}}}_{\text{X}}$	MCB	DSC	UNC
ENS	0.266	0.066	0.044	0.244
EPC	0.234	0.022	0.032	0.244
EMOS	0.232	0.018	0.030	0.244
Logistic	0.206	0.017	0.056	0.244

Interestingly, every proper scoring rule admits a representation as a mixture of elementary scoring rules (e.g., ref. 42, section 3.2). Consequently, the MCB, DSC, and UNC components of the CORP decomposition admit analogous representations as mixtures of the respective components under the elementary scores, whence we may plot Murphy diagrams in the sense of Ehm et al. (46).

## Discussion

Our paper addresses two long-standing challenges in the evaluation of probabilistic classifiers by developing the CORP reliability diagram that enjoys theoretical guarantees, avoids artifacts, allows for uncertainty quantification, and yields a fully automated choice of the underlying binning, without any need for tuning parameters or implementation choices. The associated CORP decomposition disaggregates the mean score under any proper scoring rule into components that are guaranteed to be nonnegative.

Of particular relevance is the remarkable fact that CORP reliability diagrams feature optimality properties in both finite-sample and large-sample settings. Asymptotically, the PAV-(re)calibrated probabilities, which are plotted in a CORP reliability diagram, minimize estimation error, while in finite samples, PAV-calibrated probabilities are optimal in terms of any proper scoring rule, subject to the regularizing constraint of isotonicity.

**Fig. 5.**
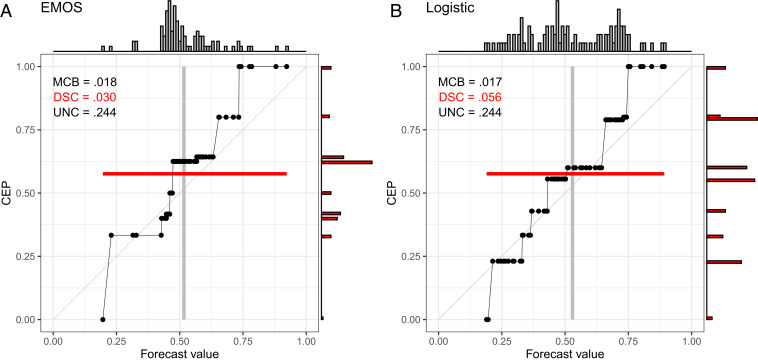
CORP discrimination diagrams for probability of precipitation forecasts over Niamey, Niger (7), in July–September 2016 with the EMOS (*A*) and Logistic (*B*) methods. The histograms at the top show the marginal distribution of the original forecast values, and the histograms at the right are for the PAV-recalibrated probabilities.

While CORP reliability diagrams are intended to assess calibration, a variant—the CORP “discrimination diagram”—focuses attention at discrimination, by adding histograms for both the original and the PAV-recalibrated forecast probabilities, as detailed in *SI Appendix*, section S6. In Fig. 5, we show examples for the EMOS and Logistic forecasts from Figs. 1 and 2 and Table 2. While both forecasts are quite well calibrated, with nearly equal Brier-score MCB components of 0.018 and 0.017, the Logistic forecast exhibits considerably higher discrimination ability, as reflected by the stronger dispersion in the vertical histogram for the PAV-recalibrated probabilities and a DSC component of 0.056, as opposed to 0.030 for the EMOS forecast. In typical current practice, discrimination ability is assessed via receiver operating characteristic (ROC) curves (47), and for a visual comparison of competing probability forecasts, ROC curves are plotted along with reliability diagrams (e.g., ref. 48). CORP discrimination diagrams offer an alternative, less directly interpretable, but more compact way of visualizing reliability and discrimination ability jointly.

We believe that the proposals in this paper can serve as a blueprint for the development of novel diagnostic and inference tools for a very wide range of data-science methods. As noted, the popular Hosmer–Lemeshow goodness-of-fit test for logistic regression is subject to the same types of ad hoc decisions on binning schemes, and hence the same types of instabilities as the binning and counting approach. Tests based on CORP and the MCB miscalibration measure are promising candidates for powerful alternatives.

Perhaps surprisingly, the PAV algorithm and its appealing properties generalize from probabilistic classifiers to mean, quantile, and expectile assessments for real-valued outcomes (49). In this light, far-reaching generalizations of the CORP approach apply to binary regression in general, to standard (mean) regression, where they yield a mean squared error (MSE) decomposition with desirable properties, and to quantile and expectile regression. In all these settings, score decompositions have been studied (45, 50), and we contend that the PAV algorithm ought to be used to generate the (re)calibrated forecast in the general decomposition in Eq. **3**, whereas the reference forecast ought to be the respective marginal, unconditional event frequency, mean, quantile, or expectile. We leave these extensions to future work and encourage further investigation from theoretical, methodological, and applied perspectives.

## Appendix A: Simulation Settings

**Fig. 6.**
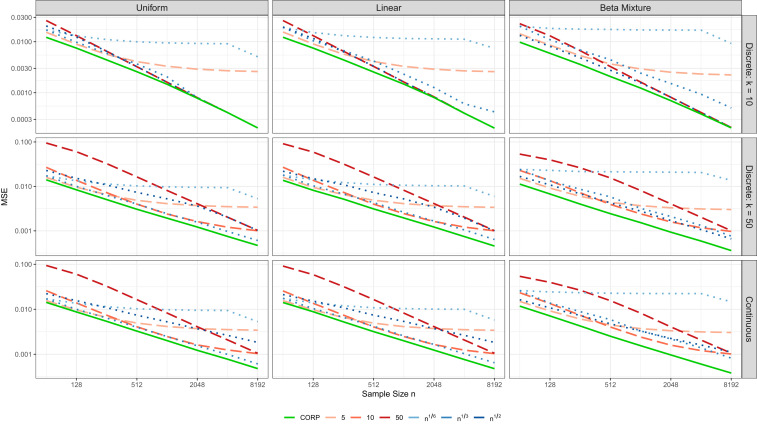
MSE of the CEP estimates in CORP reliability diagrams for samples of size n, in comparison to the binning and counting approach with m=5, 10, or 50 fixed bins, or $m\left(n\right)=\left[n^{\alpha }\right]$ quantile-based bins, where $\alpha =\frac{1}{6}$, $\frac{1}{3}$, or $\frac{1}{2}$. Note the log–log scale. The simulation settings are described in *Appendix A*, and MSE values are averaged over 1,000 replicates.

Here, we give details for the simulation scenarios in Figs. 4 and 6, where we use simple random samples with forecast values drawn from either Uniform, Linear, or Beta Mixture distributions, in either the continuous setting or discrete settings with k=10, 20, or 50 unique forecast values. The binary outcomes are drawn under the assumption of calibration, whence the true CEP function coincides with the diagonal.

We begin by describing the continuous setting, where the Uniform distribution has a uniform density and the Linear distribution a linearly increasing density with ordinate 0.40 at x=0 and 1.60 at x=1. The Beta Mixture distribution uses Beta(1,10) and Uniform components with weights $\frac{3}{4}$ and $\frac{1}{4}$, respectively. In the discrete settings with k unique forecast values, we maintain the shape of these distributions, but discretize. Specifically, for j=1,…,k, the probabilistic classifier or forecast attains the value $x_{j}=\frac{2j-1}{2k}$ with probability $p_{j}=q\left(x_{j}\right)\sum_{i=1}^{k}q\left(x_{i}\right)$, where q is the density in the continuous case. In Fig. 4, we consider discrete settings with k=10, 20, and 50 unique forecast values and the continuous case (marked Inf). Fig. 6 uses discrete settings with k=10 and 50 unique forecast values and the continuous case.

## Appendix B: Statistical Efficiency of CORP

Suppose that we are given a simple random sample $\left(x_{1},y_{1}\right),\ldots ,\left(x_{n},y_{n}\right)$ of predictive probabilities $x_{1},\ldots ,x_{n}\in \left[0,1\right]$ and associated realizations $y_{1},\ldots ,y_{n}\in \left\{0,1\right\}$ from an underlying population, with the true CEP being nondecreasing.

In the case of discretely distributed forecasts that attain a small number k of distinct values only, results of El Barmi and Mukerjee (27) imply that the MSE of the estimates in a CORP reliability diagram decays at the standard rate of $n^{-1}$. If the binning and counting approach separates the distinct forecast values, the traditional reliability diagram and the CORP reliability diagram are asymptotically the same, and so are the respective asymptotic distributions. However, under the CORP approach, the unique forecast values are always correctly identified as the sample size increases, while under the binning and counting approach, this may or may not be the case, depending on implementation decisions.

Large-sample theory for the continuously distributed case is more involved and generally assumes that the CEP is differentiable with strictly positive derivative. Asymptotic results of Wright (28) for the variance and of Dai et al. (52) for the bias imply that the MSE of the CORP estimates decays like $n^{-2/3}$. We now compare to the binning and counting approach, either using m fixed, equidistant bins or using m=m(n) empirical quantile-based bins. For a general sequence of m(n) bins, the magnitudes of the asymptotic variance and squared bias are governed by the most sparsely populated bin, at a disadvantage relative to the quantile-based case.

The classical reliability diagram relies on a fixed number m of bins, finds the respective bin-averaged event frequencies, and plots them against the bin midpoints or bin-averaged forecast values. Any such approach fails asymptotically, with estimates that are, in general, biased and inconsistent. More adequately, a flexible number m(n) of bins can be used, with boundaries defined via empirical quantiles of $x_{1},\ldots ,x_{n}$. Specifically, m(n) bins can be bracketed by zero, the empirical quantiles at level j/m(n) for j=1,…,m(n)−1, and one. Then, for n sufficiently large, each bin covers about n/m(n) data points, and the bin-averaged CEPs converge to the true CEPs at the respective true quantiles with an estimation variance that decays like m(n)/n and a squared bias that decays like $m{\left(n\right)}^{-2}$. When m(n) is of order $n^{\alpha }$ for α∈(0,1), we obtain a consistent estimate with an estimation variance that decays like $n^{\alpha -1}$ and a squared bias that decays like $n^{-2\alpha }$. Consequently, the MSE of the estimates is of order $n^{\beta }$, where β=max(α−1,−2α). The optimal choice of the exponent, $\alpha =\frac{1}{3}$, results in an MSE of order $n^{-2/3}$. While this asymptotic rate is the same as under the CORP approach, the CORP reliability diagram is preferable in finite samples, as we now demonstrate.

In Fig. 6, we detail a comparison of CORP reliability diagrams to the binning and counting approach with either a fixed number m of bins, or $m=m\left(n\right)=\left[n^{\alpha }\right]$ empirical-quantile dependent bins, where [x] denotes the smallest integer less than or equal to x∈R. For this, we plot the empirical MSE of the various CEP estimates against the sample size n, using settings described in *Appendix A*. Across columns, the distributions of the forecast values differ in shape, across rows, we are in the discrete setting with k=10 and 50 unique forecast values, and in the continuous setting, respectively. Throughout, the CORP reliability diagrams exhibit the smallest MSE, uniformly over all sample sizes and against all alternative methods, with the superiority being the most pronounced under nonuniform forecast distributions with many unique forecast values, as frequently generated by statistical or machine-learning techniques. The data-driven simulation experiments in *SI Appendix*, section S4*B* confirm the superiority of the CORP approach in terms of estimation efficiency. Only for simulation settings with nearly horizontal true CEPs, the efficiency of the CORP approach is slightly inferior to binning and counting with very small numbers of bins—exactly the choices that perform particularly poorly in almost any other setting.

## Appendix C: Properties of CORP Score Decomposition

Consider data $\left(x_{1},y_{1}\right),\ldots ,\left(x_{n},y_{n}\right)$ in the form of probability forecasts and binary outcomes, so that $x_{1},\ldots ,x_{n}\in \left[0,1\right]$, and $y_{1},\ldots ,y_{n}\in \left\{0,1\right\}$. Let ${\bar{\text{S}}}_{\text{X}}$, ${\bar{\text{S}}}_{\text{C}}$, and ${\bar{\text{S}}}_{\text{R}}$ denote the mean scores for the original forecast values, (re)calibrated probabilities, and a reference forecast, as defined in Eqs. **1** and **2**, and recall the definition of a calibrated forecast from Eq. **4**. With the specific choices of the PAV-calibrated probabilities as the (re)calibrated forecasts ${\hat{x}}_{1},\ldots ,{\hat{x}}_{n}$, and the marginal event frequency $ȳ=\frac{1}{n}\sum_{i=1}^{n}y_{i}$ as the constant reference forecast r, the score decomposition in Eq. **3** enjoys the following properties.

### Theorem 1.

*Given any set of original forecast values and associated binary events*, *suppose that we apply the PAV algorithm to generate a* (*re*)*calibrated forecast and use the marginal event frequency as reference forecast. Then*, *for every proper scoring rule* S, *the decomposition defined by* *Eqs.* **2** *and* **3** *satisfies the following*:

1) $\text{MCB}={\bar{\text{S}}}_{\text{X}}-{\bar{\text{S}}}_{\text{C}}\geq 0$ *with equality if the original forecast itself is calibrated*.
2) MCB>0 *if the score is strictly proper and the original forecast is not calibrated*.
3) $\text{DSC}={\bar{\text{S}}}_{\text{R}}-{\bar{\text{S}}}_{\text{C}}\geq 0$ *with equality if th*e (*re*)*calibrated forecast is constant*.
4) DSC>0 *if the score is strictly proper and the* (*re*)*calibrated forecast is not constant*.
5) *The decomposition is exact*.

For further discussion see *SI Appendix*, section S5, where part *A* provides the proofs of Theorems 1 and 2, and part *B* illustrates that the properties 1–4 generally do not hold if recalibration methods other than isotonic regression are used. Dawid (12) introduced the score decomposition in Eq. **3** with the subtle, but important, difference that the recalibrated probabilities are obtained as the (unique) forecast-value-wise empirical event frequencies. Then, properties 1–5 of Theorem 1 are satisfied as well, and if the sequence of (unique) forecast-value-wise event frequencies is isotonic, Dawid’s decomposition and the CORP decomposition coincide. However, isotonicity is frequently violated, especially for datasets with many unique forecast values. Then, forecast-value-wise recalibration is prone to overfitting, and, as already noted by Dawid (12), smoothing methods are required to render the approach useable.

As before, let us assume that the unique forecast values $z_{1}<\cdots <z_{k}$ are issued $n_{1},\ldots ,n_{k}$ times, with $o_{1},\ldots ,o_{k}$ of these cases being events, so that $n_{1}+\cdots +n_{k}=n$ and $o_{1}+\cdots +o_{k}=nȳ$. The classical Brier-score decomposition then becomes

$${\bar{\text{S}}}_{\text{X}}=\underset{\text{REL}}{\underset{︸}{\frac{1}{n}\overset{k}{\underset{j=1}{\sum }}n_{j}{\left(\frac{o_{j}}{n_{j}}-z_{j}\right)}^{2}}}-\underset{\text{RES}}{\underset{︸}{\frac{1}{n}\overset{k}{\underset{j=1}{\sum }}n_{j}{\left(\frac{o_{j}}{n_{j}}-ȳ\right)}^{2}}}+\underset{\text{UNC}}{\underset{︸}{ȳ\left(1-ȳ\right)}},$$

where the UNC component is the same as in the CORP decomposition in Eq. **3**. Furthermore, subject to conditions that in genuinely discrete settings may be mild, the decompositions agree in full.

### Theorem 2.

*Under the Brier score*, *if the sequence* $o_{1}/n_{1},\ldots ,o_{k}/n_{k}$ *is nondecreasing*, *then* MCB=REL *and* DSC=RES, *respectively*.

## Supplementary Material

Supplementary File

## Data Availability

The probability of precipitation forecast data at Niamey, Niger, are from the paper by Vogel et al. (ref. 7, figure 2), where the original data sources are acknowledged. Precipitation forecasts and realizations data have been deposited at GitHub (https://github.com/TimoDimi/replication DGJ20). Additional data analyses, simulation studies, technical discussion, and the proofs of Theorems 1 and 2 have been relegated to *SI Appendix*. Reproduction material for both the main article and *SI Appendix*, including data and code in the R software environment (51), are available online (38, 53). Open-source code for the implementation of the CORP approach in the R language and environment for statistical computing (51) is available on CRAN (38).
