# Supplementary material for: Stable reliability diagrams for probabilistic classifiers
Source: Proc Natl Acad Sci U S A. 2021 Feb 17;118(8):e2016191118. doi: 10.1073/pnas.2016191118 (PMC7923594; doi:10.1073/pnas.2016191118)
Supplement: Supplementary File [file pnas.2016191118.sapp.pdf]

PNAS
www.pnas.org

Supplementary Information for

Stable reliability diagrams for probabilistic classifiers

Timo Dimitriadis, Tilmann Gneiting and Alexander I. Jordan

Timo Dimitriadis.
E-mail: Timo.Dimitriadis@h-its.org

This PDF file includes:

Supplementary text
Figs. S1 to S14 (not allowed for Brief Reports)
Table S1 (not allowed for Brief Reports)
SI References

Supporting Information Text

In this supporting information text we begin by introducing experimental data sets from meteorology, astrophysics, social science, and economics (Section S1). In Section S2 we draw on these data sets to provide further illustrations of the instability of reliability diagrams and numerical measure of (mis)calibration under binning and counting approaches. This is followed by a discussion of uncertainty quantification in Section S3, where we give details for the generation of consistency and confidence bands via resampling or asymptotic theory. Section S4 supplements the simulation experiments in the main article in data-driven settings. In Section S5 we provide proofs of the theorems on score decompositions in Appendix C of the main article, and we demonstrate in examples that the claimed properties may get violated if recalibration methods other than isotonic regression are used. Finally, Section S6 introduces variants and extensions of CORP reliability diagrams, to which we refer as CORP discrimination diagrams.

S1. Experimental data sets

We now describe the experimental data sets employed subsequently. Table S1 summarizes basic properties of the data sets, which have been discussed in the meteorological, astrophysical, social science, and economic literature, respectively. In the table, the first column names the data set and lists key references. The second column lists the acronyms of the probability forecasts considered, as described in detail below. The third column shows the number of forecast cases. The fourth column provides a brief description of the type of forecast, which ranges from human subjective and personal judgements to ensemble votes from numerical-physical models and, arguably the most prevalent case in practice, output from statistical and machine learning models. The fifth and sixth column show the parameter values, α_P and β_P, for a Beta probability density function (PDF) fit to the marginal distribution of the forecast values, using the maximum likelihood technique. Similarly, the final two columns show the parameter values, α_C and β_C, for a Beta cumulative distribution function (CDF) fit (1) to the respective conditional event probability (CEP), obtained by minimizing the mean Brier score for the outcomes with respect to the fitted Beta CDF values. Values of α_C and β_C close to one are indicative of probability forecasts that are close to being calibrated, as confirmed in Fig. S1, where we show the respective CORP reliability diagrams. All four data sets are included in the reproduction material for the main paper and the supplementary document (2).

Table S1. Description of Data Sets

Data Set and Key Reference(s)	Forecast	Size	Type	α_P	β_P	α_C	β_C
Precipitation at Niamey, Niger (3)	ENS	92	ensemble	1.28	0.35	0.64	0.36
	EMOS	92	postprocessed ensemble	7.82	7.23	1.97	2.24
	EPC	92	climatological	15.40	14.40	2.45	2.70
	Logistic	92	statistical/machine learning	3.89	3.45	1.62	1.82
C1 solar flares (4–6)	NOAA	731	human interactive	0.72	1.76	1.34	1.33
	DAFFS	731	statistical/machine learning	0.56	1.27	0.99	0.69
M1 solar flares (4–6)	NOAA	731	human interactive	0.56	8.47	3.37	5.19
	DAFFS	731	statistical/machine learning	0.15	3.28	1.40	1.29
Recidivism of defendants in Broward County, Florida (7, 8)	COMPAS	1000	commercial software	0.90	1.26	0.34	0.39
	MTurk	1000	human subjective	0.64	0.60	0.33	0.28
	Logit	1000	statistical/machine learning	2.37	2.93	1.30	1.50
	GBM	1000	statistical/machine learning	2.09	2.44	0.98	1.06
U.S. GDP recessions (9–11)	SPF #84, nowcast	119	personal/unknown	0.16	0.76	0.83	0.48
	SPF #84, 1Q ahead	121	personal/unknown	0.34	1.59	0.75	0.52
	SPF #84, 4Q ahead	118	personal/unknown	2.74	10.60	0.15	0.03
	SPF avg., nowcast	203	survey mean	0.71	2.66	1.33	1.28
	SPF avg., 1Q ahead	202	survey mean	1.42	5.88	1.45	1.72
	SPF avg., 4Q ahead	195	survey mean	7.50	35.90	0.31	0.11

A. Precipitation at Niamey, Niger. This data set of 24-hour ahead daily probability of precipitation forecasts at Niamey, Niger in July—September 2016 has been described and utilized in the main article already. To recall, we consider forecasts from the following four methods: the internationally leading 52-member numerical weather prediction (NWP) ensemble system run by the European Centre for Medium-Range Weather Forecasts (ENS), a statistically postprocessed version of the latter called ensemble model output statistics (EMOS), a reference forecast called extended probabilistic climatology (EPC), and a purely data-driven statistical forecast (Logistic). The respective CORP reliability diagrams are shown and discussed in the main article, and for completeness we include them in panel (a) of Fig. S1 as well. Further information can be found in the original paper by Vogel et al. (3), where the data studied here are visualized in Fig. 2.

B. Solar flares. Solar flares disrupt radar and terrestrial communications systems in the sunlit hemisphere. Recently, the scientific understanding of solar flares has increased to the point that multiple operational forecasts have become available, as compared and evaluated by Barnes et al. (12) and Leka et al. (4). We consider probability forecasts for solar flares at two magnitudes, denoted C1 and M1, respectively, that correspond to the binary event of a flare exceeding thresholds of 10^{-6} and

(a) Precipitation at Niamey, Niger

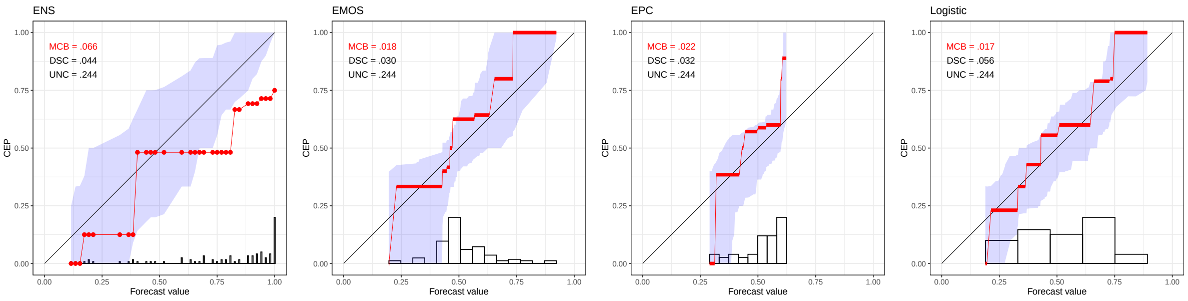

(b) Solar flares

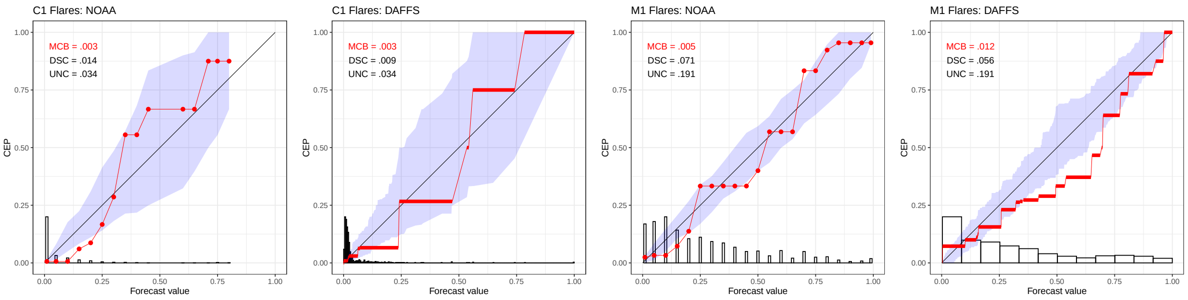

(c) Recidivism of defendants in Broward County, Florida

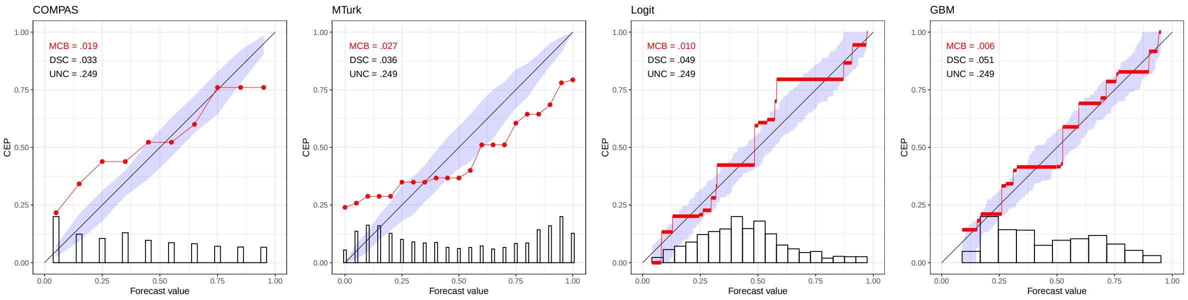

(d) U.S. GDP recessions

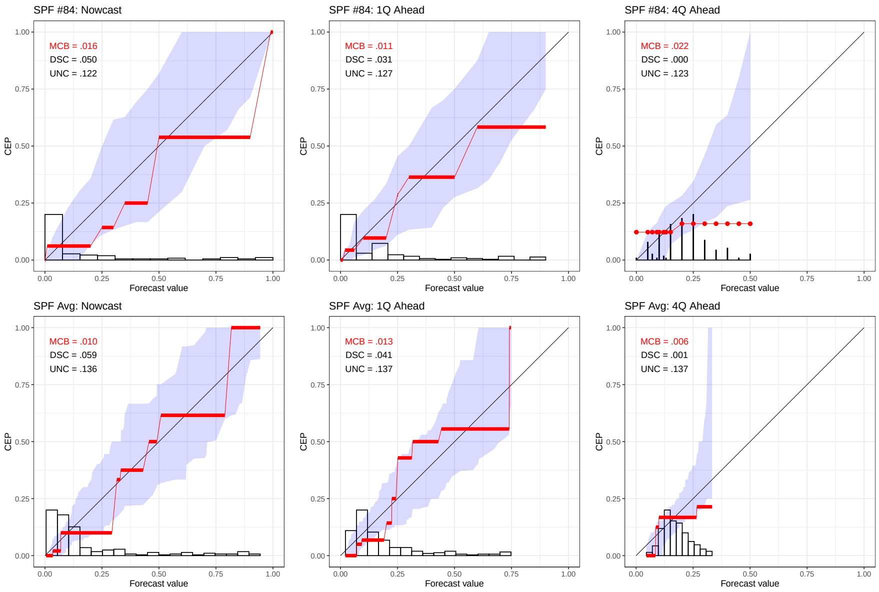

Fig. S1. CORP reliability diagrams with 90% consistency bands for the probability forecasts in the four data sets described in Table S1.

10^{-5} Wm^{-2} within the next 24 hours (4). Specifically, we compare human interactive forecasts issued by the National Oceanic and Atmospheric Administration Space Weather Prediction Center (NOAA; 5), which are widely considered as a benchmark (4), and by NorthWest Research Associates' Discriminant Analysis Flare Forecasting System (DAFFS; 6) for the period from January 1, 2016 to December 31, 2017. As Fig. S1(b) shows, these forecasts are reasonably well calibrated, except for M1 DAFFS. Forecast probabilities and binary occurrence data are available online (13).

C. Recidivism of defendants in Broward County, Florida. In the U.S. criminal justice system, algorithms have been used to assess the probability that criminal defendants re-offend (7). The Correctional Offender Management Profiling for Alternative Sanctions (COMPAS) software tool was designed for this task and predicts the likelihood (through an integer score between 1 and 10) of committing either a misdemeanor or a felony in the following two years, based on 137 features of defendants and their crime history. The use of this type of tool has triggered vigorous debate about the fairness of algorithms. Here we utilize a database of pre-trial defendants from Broward County, Florida (14) that comprises 7214 cases from 2013 and 2014. In order to be able to train methods other than COMPAS, we base our analysis on the 1000 defendants drawn randomly by Dressel and Farid (7). We transform the COMPAS integer scores $s \in \{1, 2, \ldots, 10\}$ into probabilities $p \in [0, 1]$ through the crude formula $p = (s - .5)/10$, resulting in equally spaced values between .05 and .95. Dressel and Farid (7) further consider predictions made by lay people with little or no criminal justice expertise, recruited through Amazon's Mechanical Turk, an online platform where participants get hired to perform a wide variety of tasks. We refer to their paper for details on forming these probability forecasts, which we denote by MTurk. Bansak (8) employs simple statistical/machine learning procedures, logistic regression (Logit) and gradient boosted trees (GBM), to forecast the recidivism probability, based on training data. In Fig. S1(c) we see that the statistical/machine learning based techniques yield quite well calibrated probabilities, whereas the MTurk forecasts lack calibration. The original data are available at https://doi.org/10.7910/DVN/KT20FE.

D. U.S. GDP recessions. The Survey of Professional Forecasters (SPF; 9, 10) assembles probability forecasts of real U.S. gross domestic product (GDP) decline for the current quarter (so-called nowcasts), as well as for the subsequent four quarters. The individual forecasts are collected in real time from anonymous economic experts, who are identified by ID number. This is a well studied data set in macroeconomics; e.g., Lahiri and Wang (11) study the quality of the forecasts via quadratic and logarithmic probability scores, through receiver operating characteristic (ROC) curves, and by assessing calibration. We consider nowcasts along with one quarter (1Q) and four quarters (4Q) ahead forecasts from the survey participant who issued the most forecasts over time, namely ID #84, and from the average (avg.) forecast, in the sense of the equally weighted mean over the participants who had contributed probability forecasts for the target under consideration. Forecaster ID #84 issued predictions from the fourth quarter of 1968 through the fourth quarter of 2009, and the avg. forecast was available to us for issue dates from the fourth quarter 1968 through the second quarter of 2019 (nowcasts and one quarter ahead) and the second quarter of 2018 (four quarters ahead), respectively. As Fig. S1(d) shows, nowcasts and one quarter ahead forecasts are reasonably well calibrated. However, probability forecasts at a prediction horizon of four quarters ahead lack discrimination ability. The original forecast data are available at https://www.philadelphiafed.org/surveys-and-data/recess, and the corresponding realizations can be downloaded at https://www.philadelphiafed.org/surveys-and-data/real-time-data-research/routput.

S2. (In)stability: Illustrative examples

Here we illustrate in further detail that reliability diagrams and quantitative measures of (mis)calibration generated under binning and counting schemes exhibit unwarranted instabilities.

A. Reliability diagrams. In the main paper, Fig. 2 demonstrates instability with respect to the number of bins used under the popular choice of equidistant binning. Here we strengthen the argument by also considering *quantile-based* binning, which is arguably the most natural approach for stabilization in binning and counting schemes. Perhaps surprisingly, while quantile-based approaches tend to improve stability, the resulting reliability diagrams may still depend heavily on the number of bins as well as on implementation details. In contrast, the CORP approach yields a unique, stable solution without any need for implementation decisions.

We consider three natural implementations of quantile-based binning, all using a nominal number of m bins, and refer to them as Q, Q$^+$ and Q$^-$ variants, respectively:

Variant Q We find the sample quantiles at level $1/m, \ldots, (m-1)/m$ of the forecast values, using the default version of the `quantile()` function in R (15). Then, we form m bins by taking the sample quantiles together with 0 and 1 as bin breaks, where the lower end of the interval is closed while the upper end is open, except for the last bin. Multiple occurrences of the same bin break are ignored, resulting in the possible use of less than m bins.

Variant Q$^+$ We sort the forecast–realization pairs by their forecast values. In the case of ties in the forecast value, we sort by outcome values in *ascending* order. Then we place the cases in m equally populated bins, based on this order. If the size of the data set is not a multiple of m, excess values are redistributed in such a way that the bins with an additional observation are as far apart from each other as possible.

Variant Q$^-$ Same as variant Q$^+$, except that in the case of ties in the forecast value we sort in *descending* order of the binary outcome values.

Figs. S2–S5 show reliability diagrams generated under the binning and counting approach with quantile-based bins in the Q, Q^+, and Q^- variants and reliability diagrams generated under binning and counting with equidistant bins, where each variant is employed with $m = 9, 10$, and 11 bins. We furthermore show the respective CORP reliability diagram, which is unique and independent of implementation decisions. Each figure concerns a single forecast described in Table S1.

In Fig. S2 we return to the ENS probability of precipitation forecast at Niamey, Niger in Fig. 1 of the main article. Note that panels (a) and (b) in the latter are recovered by the left-hand diagram in the second row and the diagram in the fourth row of Fig. S2, respectively. The unconditional frequency of precipitation in this data set is 57.6%. Many forecasts have values close to one, with a total of 24 out of 92 having the exact value of one, corresponding to cases where all 52 members of the ECMWF forecast ensemble show rain over Niamey. This results in considerable instability of the quantile-based reliability diagrams at and near a forecast value of one with respect to both the binning variant employed and the number of bins used. In contrast, the CORP reliability diagram stabilizes estimates in this region.

Fig. S3 shows reliability diagrams for the DAFFS forecast of solar flares at magnitude M1. Here we have an unconditional event frequency of 3.6% only, and a vast majority of probability forecasts is at values below .05, challenging the visual assessment of calibration under the binning and counting approach. Importantly, small unconditional event frequencies are common in disciplines such as meteorology or solar physics, where interest frequently focuses on predictions of rare and extreme events. In such settings, equidistant binning tends to incur instabilities driven by sparsely populated upper end bins, as observed in this example. While all nine versions of quantile-based reliability diagrams indicate calibrated forecasts, there is considerable instability in the estimated CEP curves' shapes. Notably, the Q variant generates curves up to a forecast value of almost one, whereas the Q^+ and Q^- variants stop at about .25. For all instances, the concentration of bin breaks at very small values hinders visual assessment. In contrast, the CORP reliability diagram shows the estimated CEP curve on the full range of actual forecast values. The estimate itself is stable, and the aforementioned issues get reflected differently, namely, in consistency bars that are narrow for low forecast values, and getting broader for higher forecast values.

Fig. S4 turns to reliability diagrams for the (linearly transformed) COMPAS score for the probability of recidivism of criminal defendants. Here we note an unconditional event frequency of 47.6% and a relatively balanced, though slightly skewed distribution of the forecast values on the unit interval. For this nicely behaved data set, both equidistant and Q binned reliability diagrams are stable. However, the Q^+ and Q^- variants display instabilities with respect to both the method chosen and the number of bins.

Finally, Fig. S5 shows reliability diagrams for SPF participant #84 and probability forecasts of a decline in U.S. GDP at a prediction horizon of four quarters ahead. The data set exhibits an unconditional event frequency of 14.4% and the forecast values are all below .50, with a vast majority being below .25. The reliability diagrams generated under the binning and counting approach deliver an unclear message, with estimates of CEPs showing zigzag patterns, reflecting estimation noise. In contrast, the CORP reliability diagram shows a nearly constant estimate of the CEP curve, indicating that the forecasts fail to distinguish between periods with high and low probability of a GDP decline. This is unsurprising as forecasting GDP a year ahead is an inherently difficult problem. As before, we note that CORP reliability diagrams reflect high estimation uncertainty and challenging forecasting problems with low signal to noise ratios in broad consistency bands, while regularizing the estimated CEP curve itself.

B. Numerical measures of (mis)calibration. As pointed out in the main paper, instabilities in reliability diagrams generated under binning and counting approaches propagate to associated numerical measures of (mis)calibration. To illustrate this, the upper left corners of the plots in Figs. S2–S5 show associated Brier score components. For the reliability diagrams generated under the binning and counting approach, we follow extant practice and report the REL, RES, and UNC components of the classical bin-sensitive Brier score decomposition, as reviewed by (16), that honors the binning in the plot at hand. For the CORP method, we report the values of the MCB, DSC, and UNC components in our proposed CORP Brier score decomposition, as introduced in the main article and discussed in more detail in Section S5 below.

Under the binning and counting approach, we throughout find instabilities of the REL and RES components with respect to both the binning method chosen and the number of bins, in ways comparable to the instabilities found in the previous subsection. For instance, in the SPF example in Fig. S5 participant #84 achieves a mean Brier score of .145, and the marginal event frequency is 14.4%, for a shared UNC component of .123. However, the values of the REL component range from .021 to .053, and the values of the RES component from .002 to .026, across the twelve variants of binning and counting. For comparison, the CORP decomposition yields MCB = .0224 and DSC = .0003, assigning the bulk of the mean score to the UNC component, well in line with our above interpretation.

As hinted at in the main article, instabilities of numerical measures of (mis)calibration that derive from binning and counting have been reported before, namely in the context of the Hosmer-–Lemeshow goodness of fit test in binary regression (17), where the focus is on hypothesis testing, rather than visualization. In a nutshell, for the Hosmer–Lemeshow test the fitted probabilities from a parametric binary regression model assume the role of the classifier values or probability forecasts, and the goal of the test is to check whether the binary regression model is correctly specified in terms of user decisions such as the functional form, the link function, and the choice of predictor variables. The test is carried out by assessing calibration of the system of fitted probabilities $x_1, \ldots, x_n$ and associated binary outcomes $y_1, \ldots, y_n$. Adopting the binning and counting approach, the test procedure places $x_1, \ldots, x_n$ in m (either equidistant, or quantile-based) mutually exclusive bins, labeled by indices $j = 1, \ldots, m$. Borrowing notation from Stephenson et al. (16), for bin j, we denote the average predicted probability by $\bar{x}_j$, the average event frequency by $\bar{y}_j$, and the bin-specific sample size by n_j. The Hosmer–Lemeshow test statistic HL and the

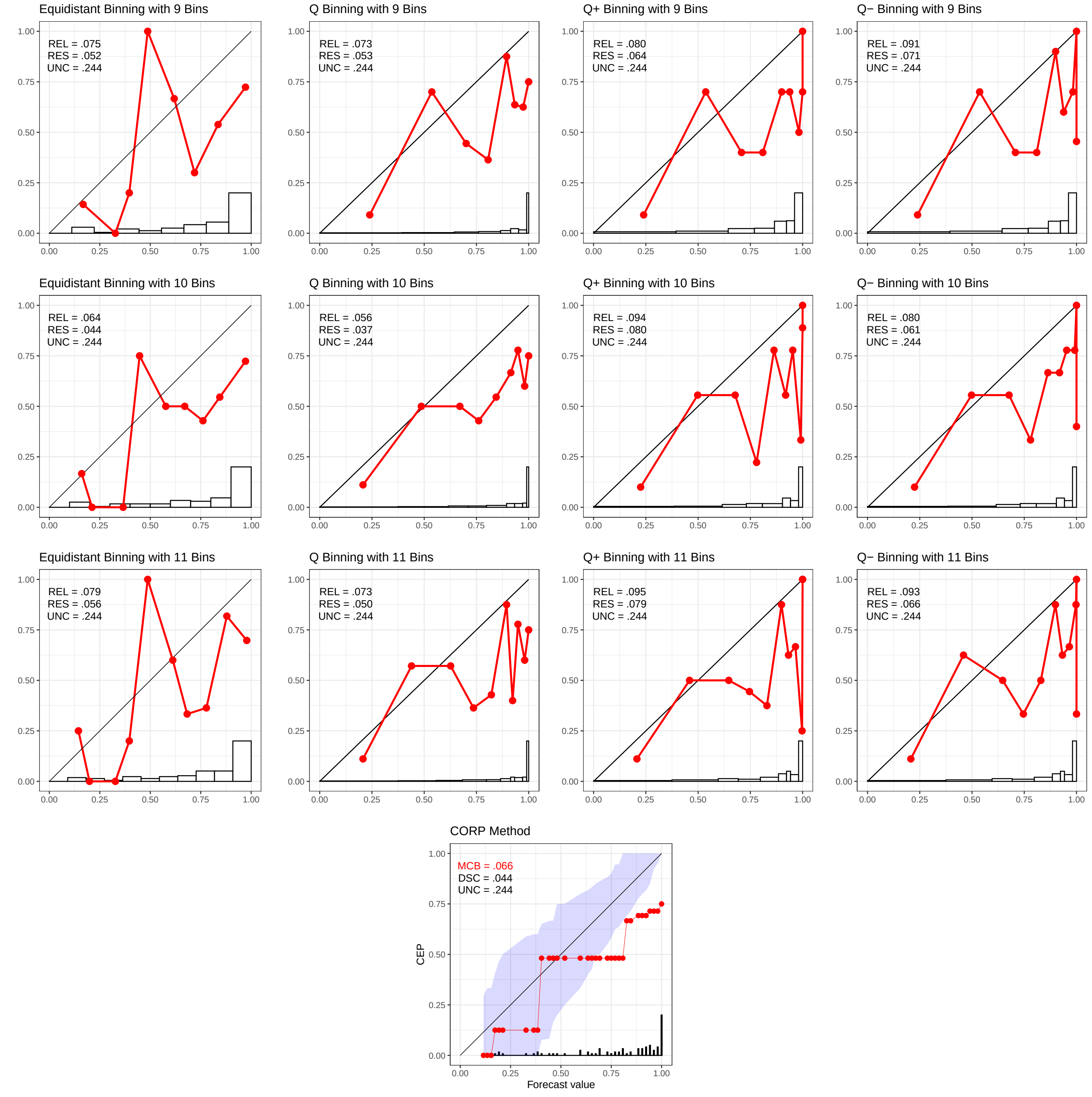

Fig. S2. Comparison of reliability diagrams generated under the binning and counting approach to the CORP reliability diagram, for the ENS probability of precipitation forecast at Niamey, Niger. The upper three rows show reliability diagrams under the binning and counting paradigm, based on distinct methods for bin selection (from left to right: equidistant, Q, Q^+, Q^-) and using (from top to bottom) $m = 9, 10,$ and 11 bins, respectively. The bottom row shows the CORP reliability diagram with 90% consistency bands.

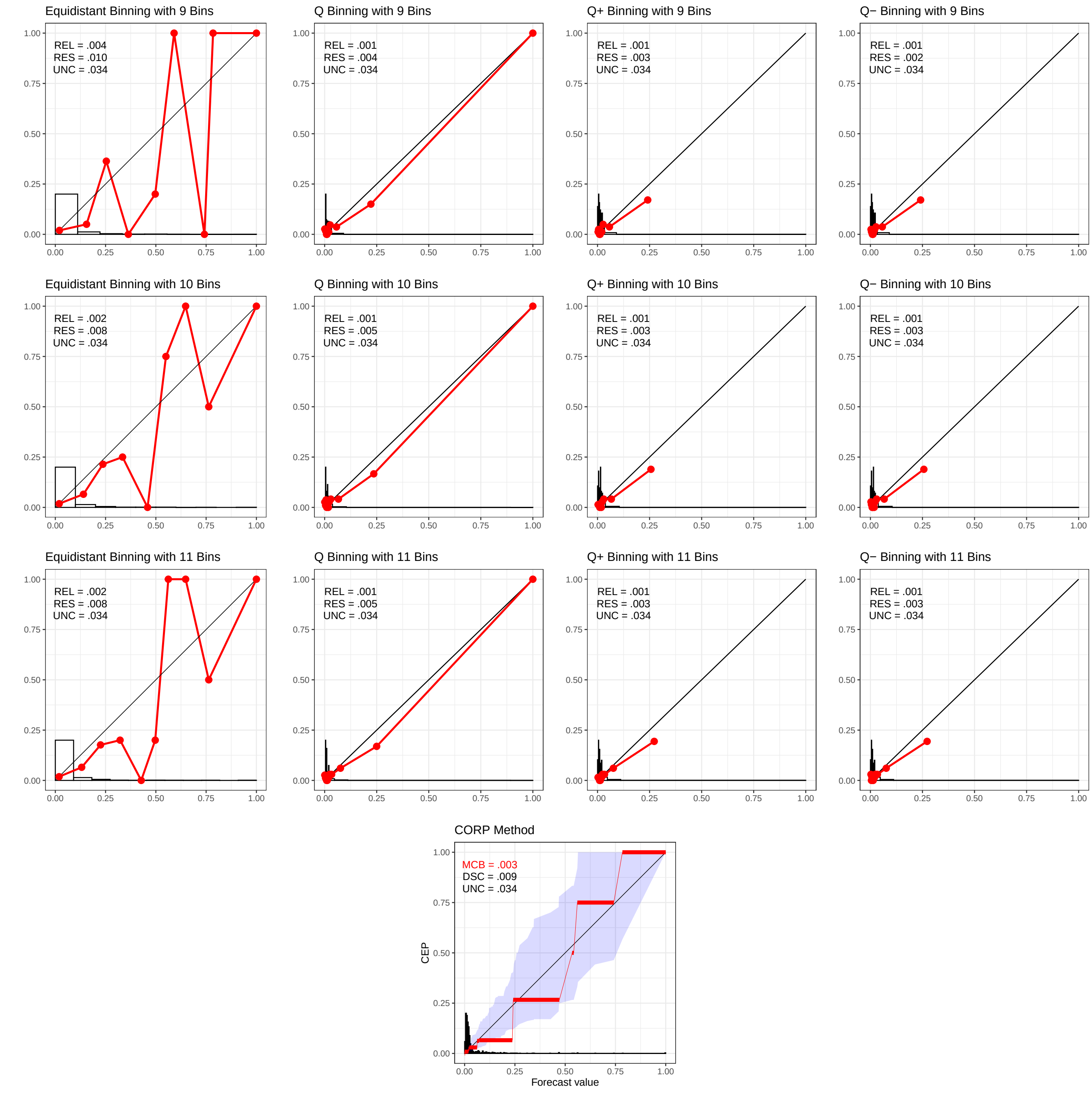

Fig. S3. Same as Fig. S2, but for DAFFS probability forecasts of M1 solar flares.

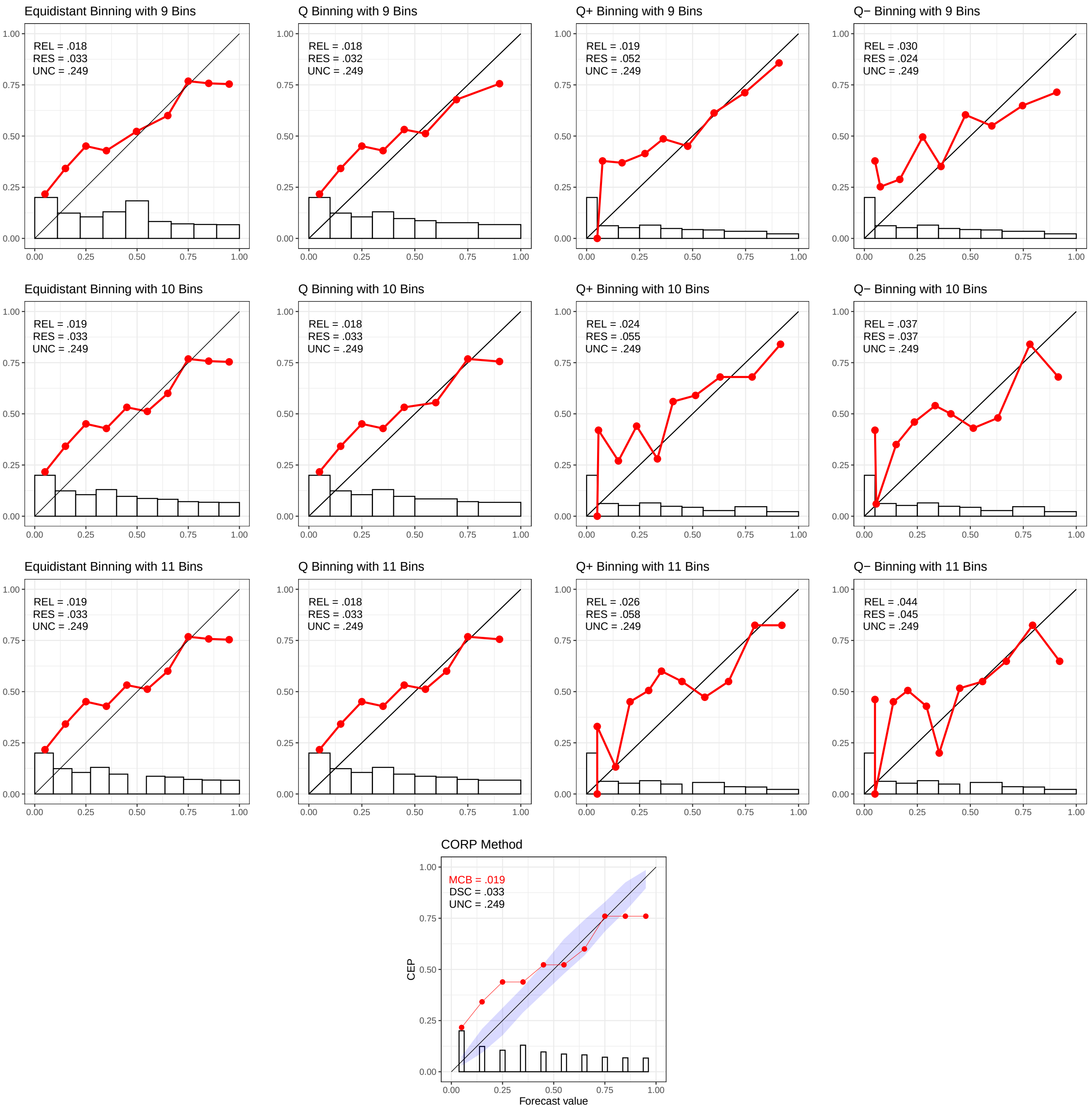

Fig. S4. Same as Fig. S2, but for COMPAS scores for recidivism.

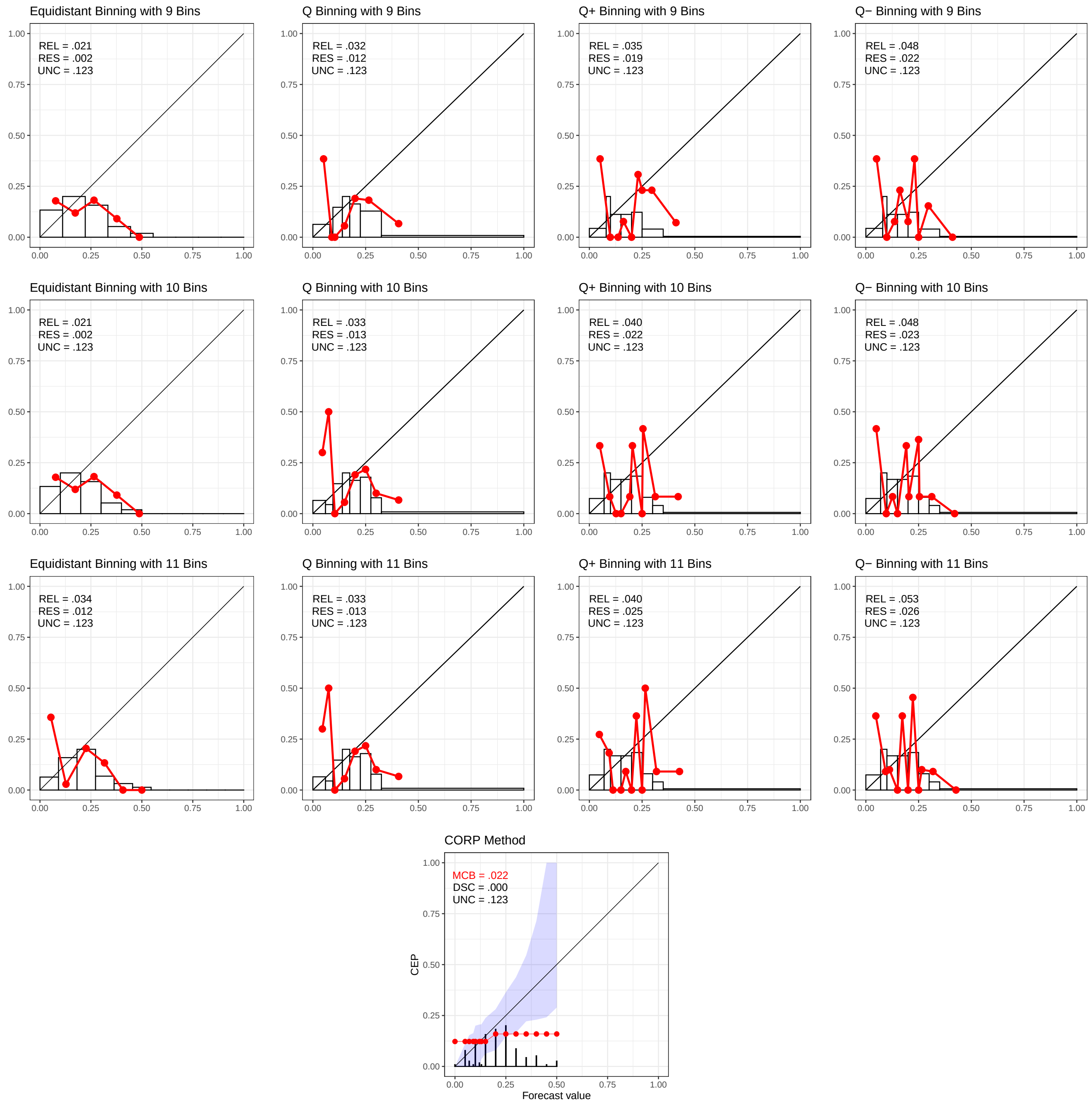

Fig. S5. Same as Fig. S2, but for four quarters ahead probability forecasts of U.S. GDP decline by SPF participant #84.

aforementioned REL component of the classical Brier score decomposition then compare as

$$\mathsf{HL} = \sum_{j=1}^{m} n_j \frac{(\bar{y}_j - \bar{x}_j)^2}{\bar{x}_j(1-\bar{x}_j)} \qquad \text{and} \qquad \mathsf{REL} = \frac{1}{n}\sum_{j=1}^{m} n_j (\bar{y}_j - \bar{x}_j)^2. \tag{1}$$

Hence, the Hosmer–Lemeshow test statistic can be interpreted as a bin-wise standardized version of the REL measure. Under the null hypothesis of a correct specification of the binary regression model (i.e., perfect calibration), the term $\bar{x}_j(1-\bar{x}_j)$ serves to standardize the variance, and thus is essential for the large sample χ^2 limit of the Hosmer–Lemeshow statistic.

In this light, the REL component in the classical Brier score decomposition and the Hosmer–Lemeshow test statistic HL are subject to the same types of ad hoc decisions on binning schemes and, consequently, to the same types of instabilities. For the Hosmer–Lemeshow statistic, its lack of stability has been described and discussed in both research papers (18–20) and textbooks (21, p. 92; 22, p. 236; 23, p. 173). Perhaps most drastically, Bertolini et al. (18) observe that for mortality data from 1393 intensive care patients in Italy, the standard implementation of the 10 bin Hosmer–Lemeshow test in the SAS software is highly unstable under mere re-ordering of the very same data set: Across all possible re-orderings, p-values between .01 and .95 are observed, implying that a researcher can tailor any desired test decision to their will, in astonishing defiance of any stability and trustworthiness. In contrast, as noted in the discussion section of the main article, we anticipate that goodness of fit tests within the CORP framework (e.g., based on the MCB measure) can overcome these issues.

S3. Uncertainty quantification: Technical details

In this section we describe implementation details for the generation of pointwise consistency or confidence bands to accompany a CORP reliability diagram.

Suppose that we have a data set of size n, consisting of probability forecasts $x_1, \ldots, x_n \in [0,1]$ with unique forecast values $z_1 < \cdots < z_k$, and associated binary outcomes $y_1, \ldots, y_n \in \{0,1\}$. As noted in the main paper, our software implementation uses the following default choices. For consistency bands, if $n \le 1000$, or if $n \le 5000$ and $n \le 50k$, we use resampling, else we rely on asymptotic theory. In the latter case we employ the discrete asymptotic distribution if $n \ge 8k^2$, while otherwise we use the continuous one. For confidence bands, the default uses resampling throughout, as extant asymptotic theory depends on the assumption of a true CEP with strictly positive derivative.

The resampling technique is described in the following algorithm, where the (isotonic regression) CORP estimate of the CEP curve based on the original data $(x_1, y_1), \ldots, (x_n, y_n)$ is denoted $\widehat{\mathrm{CEP}}(\cdot)$. As noted in the main article, the PAV algorithm assigns recalibrated probabilities to the unique forecast values $z_1 < \cdots < z_k$, and to facilitate visual inspection, we interpolate linearly in between. Hence, $\widehat{\mathrm{CEP}}(\cdot)$ is a piecewise linear function that is defined on the interval $[z_1, z_k]$ only, and similarly for the resampled estimate that is defined on the range of the resampled classifier values only. In the algorithm, the first loop is over m resampling replicates, with $m = 100$ being the default and used throughout, and the second loop is over the k unique values of the original probability forecasts.

Algorithm 1 Resampling-based pointwise consistency and confidence bands at level $(1-\alpha) \times 100$ percent

for l *in* $1:m$ **do**
  sample $x_1^{(l)}, \ldots, x_n^{(l)}$ from $x_1, \ldots, x_n$ with replacement
  for i *in* $1:n$ **do**
    if *consistency bands* **then**
      draw $y_i^{(l)}$ from Bernoulli$(x_i^{(l)})$
    end
    if *confidence bands* **then**
      draw $y_i^{(l)}$ from Bernoulli$(\widehat{\mathrm{CEP}}(x_i^{(l)}))$
    end
  end
  use the resampled data set $(x_1^{(l)}, y_1^{(l)}), \ldots, (x_n^{(l)}, y_n^{(l)})$ and the PAV algorithm to find the estimate $\widehat{\mathrm{CEP}}^{(l)}(\cdot)$ at the unique values of $x_1^{(l)}, \ldots, x_n^{(l)}$, and interpolate linearly in between
end
for j *in* $1:k$ **do**
  use empirical quantiles of the non-missing resampled values among $\widehat{\mathrm{CEP}}^{(1)}(z_j), \ldots, \widehat{\mathrm{CEP}}^{(m)}(z_j)$ at level $\frac{\alpha}{2} \times 100$ and $(1-\frac{\alpha}{2}) \times 100$ percent, respectively, to obtain uncertainty bars at z_j
end
interpolate the thus obtained uncertainty bars at $z_1, \ldots, z_k$ linearly, to obtain pointwise uncertainty bands on the full range $[z_1, z_k]$ of the original forecast values

In the subsequent discussion we review extant asymptotic theory under the standard assumption that the data $(x_1, y_1), \ldots, (x_n, y_n)$ comprise independent, identically distributed draws from the joint distribution of (X, Y). For confidence bands, our defaults never revert to asymptotic theory, as noted, so we discuss consistency bands only.

In the discrete case we apply asymptotic theory developed by El Barmi and Mukerjee (24). Then the lower and upper bounds of the symmetric $(1-\alpha)\cdot 100$ % consistency bar at the unique forecast value z_j are

$$z_j \pm \left(\frac{z_j(1-z_j)}{n_j}\right)^{1/2} q_{\alpha/2}(N), \qquad [2]$$

where $q_{\alpha/2}$ is the $\alpha/2$-quantile of a standard normal random variable N. This formulation is implied by Theorem 2 in El Barmi and Mukerjee (24) under the assumption that $\mathrm{CEP}(z_j) = z_j$ for $j = 1, \ldots, k$. Then the sets $\mathcal{S}_{ix}$ in their equation (6) are singletons, and the asymptotic distribution of the max–min formulation in equation (8) simplifies to a normal distribution. Finally, we impose the obvious cut-offs of the lower and upper limits at zero and one, respectively. Between the unique forecast values, we interpolate linearly, to obtain visually pleasing consistency bands.

In the continuous case we operate under the assumption that the forecast values have an absolutely continuous distribution with a strictly positive, bounded Lebesgue density. Then the $(1-\alpha)\cdot 100$ % consistency bar at the forecast value $z_j \in [0,1]$ is obtained as

$$z_j \pm \left(\frac{z_j(1-z_j)}{2n\,\hat{f}_n(z_j)}\right)^{1/3} q_{\alpha/2}(2T), \qquad [3]$$

where $q_{\alpha/2}(2T)$ denotes the $\alpha/2$-quantile of two times a variable T with Chernoff's distribution (25), and $\hat{f}_n$ is a (boundary robust) kernel density estimate of the unconditional density of the classifier values. This formulation follows directly from Theorem 1 in Wright (26). Again, we impose the obvious cut-offs of the lower and upper limits at zero and one, respectively, and in between forecast values we interpolate linearly.

S4. Data-driven simulations

In this section, we return to the simulation examples in the main paper, but now under data-driven scenarios.

A. Uncertainty quantification: Data-driven simulations. First, we extend the simulation study on the coverage of confidence and consistency bands, as reported in Fig. 4 of the main article, to settings that are driven by the data sets described in Section S1.

As noted in Appendix A of the main paper, the simulation design in the main article uses random samples with forecast values drawn from either Uniform, Linear, or Beta Mixture distributions, in either the continuous setting, or discrete settings with $k = 10$, 20, or 50 unique forecast values. The binary outcomes are drawn under the assumption of perfect calibration, so that the true CEP function coincides with the diagonal. In the discrete settings we maintain the shape of the continuous distributions, but discretize to k unique forecast values.

In Figs. S6–S9 we maintain this general setting, but now in data-driven scenarios for both the forecast values and the true CEP functions, as implied by the meteorological, astrophysical, social science, and economic examples described in Section S1. Specifically, for each of the 18 forecasts in Table S1, the associated simulation uses forecast values drawn from Beta densities with parameter values α_P and β_P as given in the table, and the corresponding realizations are drawn either under the assumption of perfect calibration, or from an assumed CEP that equals the CDF of a Beta distribution with parameter values α_C and β_C as given in the table. These data-generating processes capture key features of the underlying data sets.

The results in Figs. S6–S9 echo what we observe in Fig. 4 of the main article. Under nearly all of the 18 considered data-generating processes, and under assumptions of either calibration or miscalibration, the coverage rates of both consistency bands and confidence bands are close to the nominal 90% level, especially for large sample sizes. In the displays, the method used predominantly for uncertainty quantification (resampling, discrete asymptotic distribution theory, or continuous asymptotic distribution theory) across the 1000 simulation replicates is indicated by the plot symbol. Notably, we observe break points in the coverage of consistency bars as the method employed for uncertainty quantification changes from resampling to the use of asymptotic theory. The most obvious inaccuracies arise in the two columns associated with the nearly horizontal simulated CEP curves for four quarters ahead forecasts of GDP recession (Figs. S1(d) and S9), a notable case that we discuss in detail in the subsequent section.

While overall the uncertainty quantification performs as expected, we encourage and anticipate improvement in future research. In particular, the asymptotic theory developed by El Barmi and Mukerjee (24) and Wright (26), which we use to obtain large sample consistency bands, can be leveraged in similar ways to obtain confidence bands. However, considerable complications arise in cases where the true CEP has points with vanishing slope. This is an issue that requires attention so that large sample theory can be applied to confidence bands as well. Furthermore, the existence of the aforementioned breakpoints in coverage motivate the development of mixed schemes, to efficiently handle large data sets with imbalanced unconditional distributions of the forecast values, where resampling might be computationally infeasible, whereas extant asymptotic theory might result in severe inaccuracies in sparsely populated regions. In such cases, in regions (of the forecast value) that contain sufficiently many cases asymptotic theory can be employed efficiently, and suitably restricted forms of resampling offer an attractive alternative in sparsely populated regions (of the forecast values). Future developments of mixed schemes depend on mechanisms for the avoidance of artifacts as one moves from one type of region into the other, but might reap the benefits of both the small sample accuracy of resampling and the computational efficiency of methods that are based on asymptotic theory.

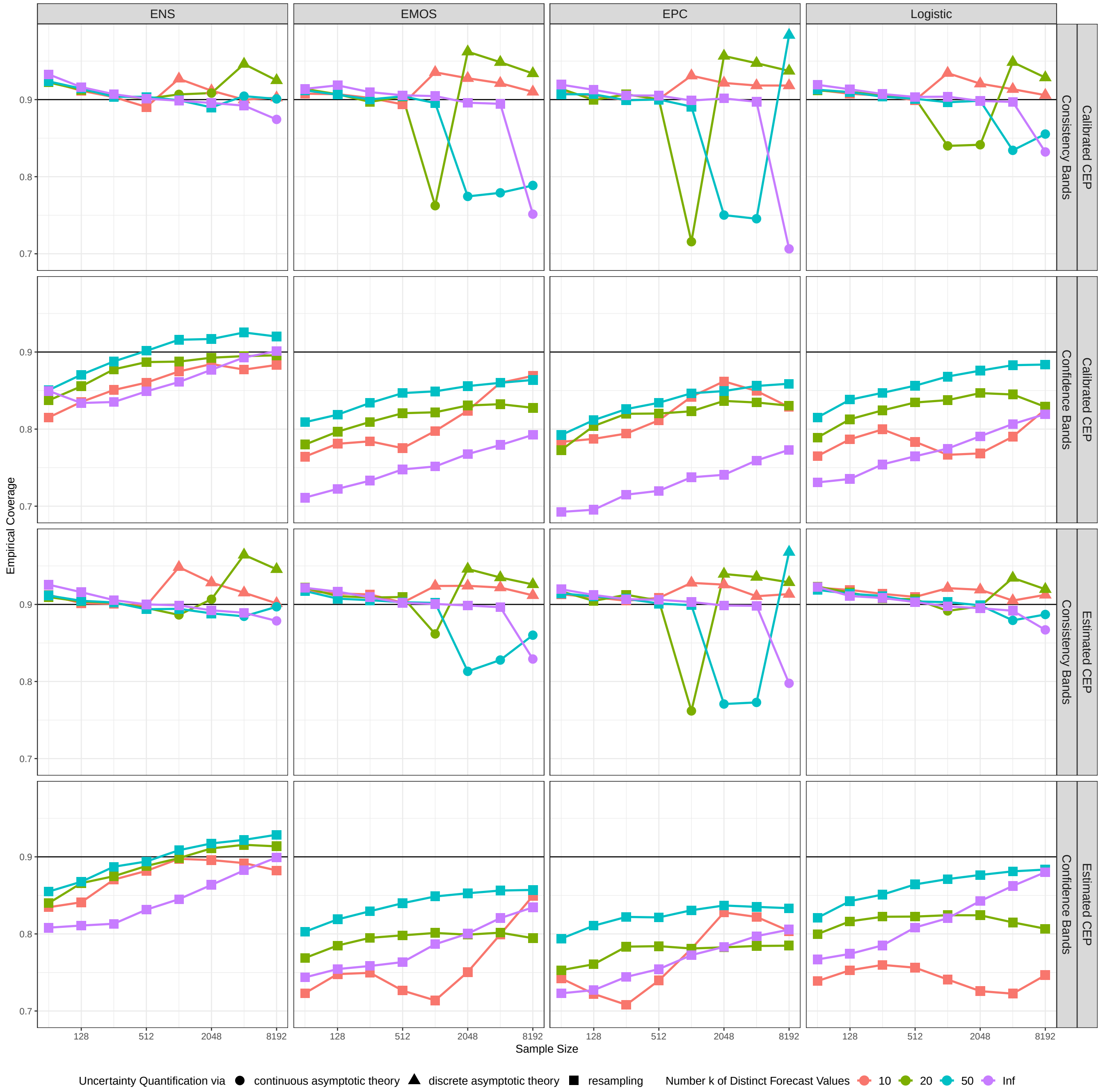

Fig. S6. Empirical coverage, averaged equally over forecast values, of 90% uncertainty bands for CORP reliability diagrams under default choices for 1000 simulation replicates. The columns correspond to Beta distributions for the forecast values with parameter values α_P and β_P associated with probability of precipitation forecasts at Niamey, Niger, as listed in Table S1. The upper two rows show results for consistency bands and confidence bands in the case of a calibrated CEP, when the binary outcomes are Bernoulli draws from the forecast probabilities. The bottom two rows show results under CEP functions that equal Beta CDFs with parameter values α_C and β_C as also listed in Table S1. For further details, see Appendix A in the main article and Part A of Section S4.

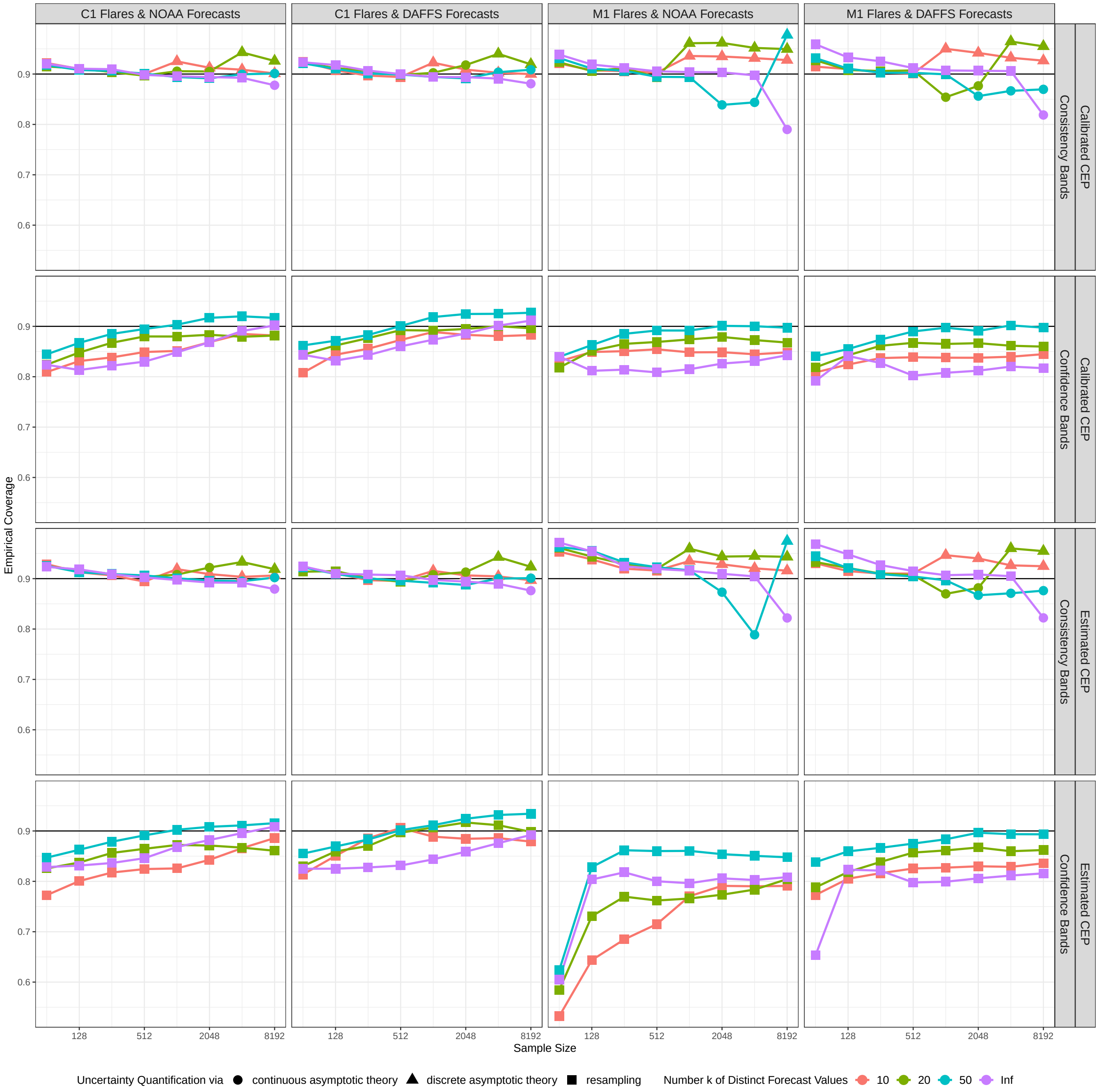

Fig. S7. Coverage rates in the setting of Fig. S6, with parameter values informed by the solar flares data described in Table S1.

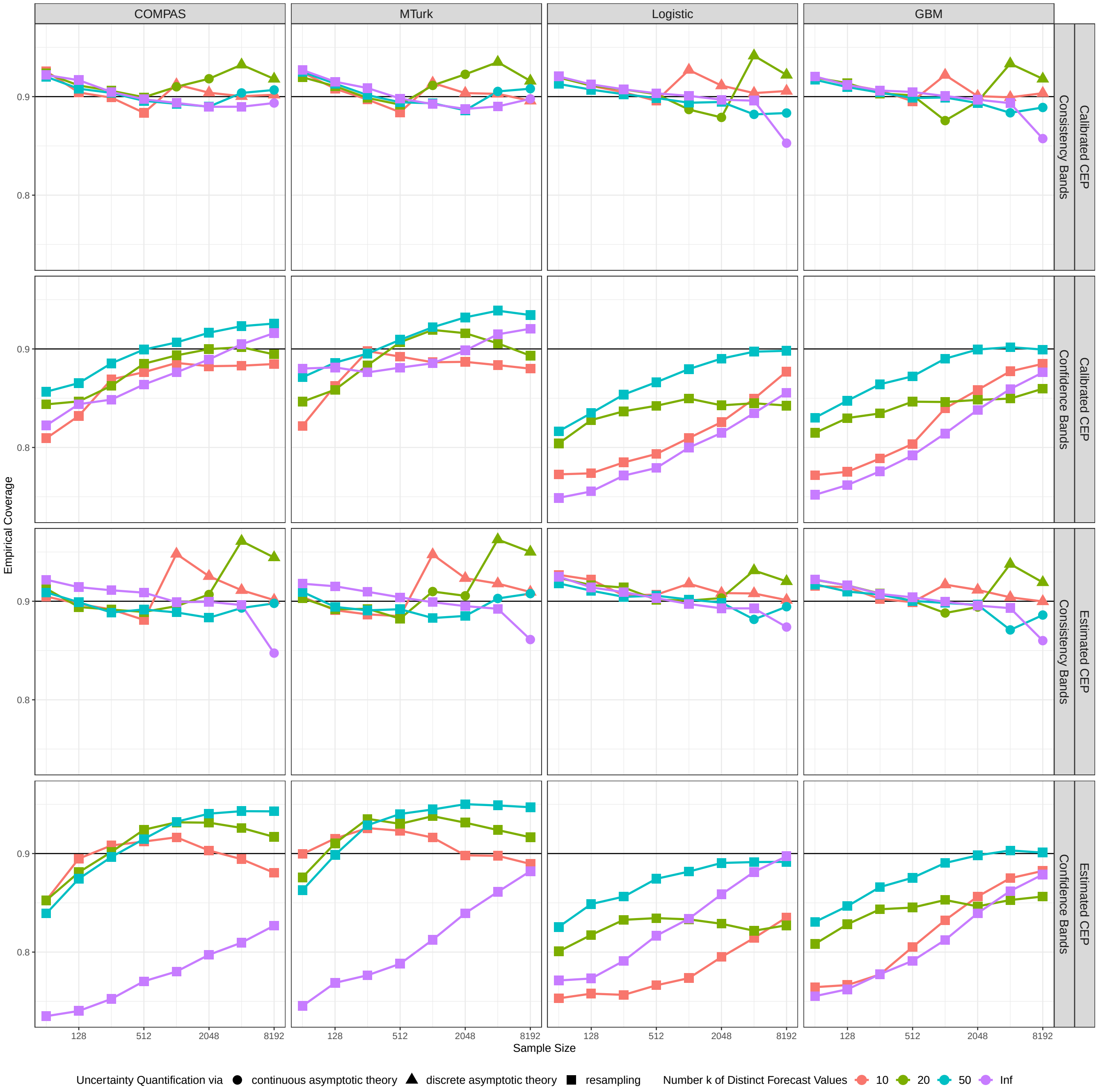

Fig. S8. Coverage rates in the setting of Fig. S6, with parameter values informed by the recidivism data described in Table S1.

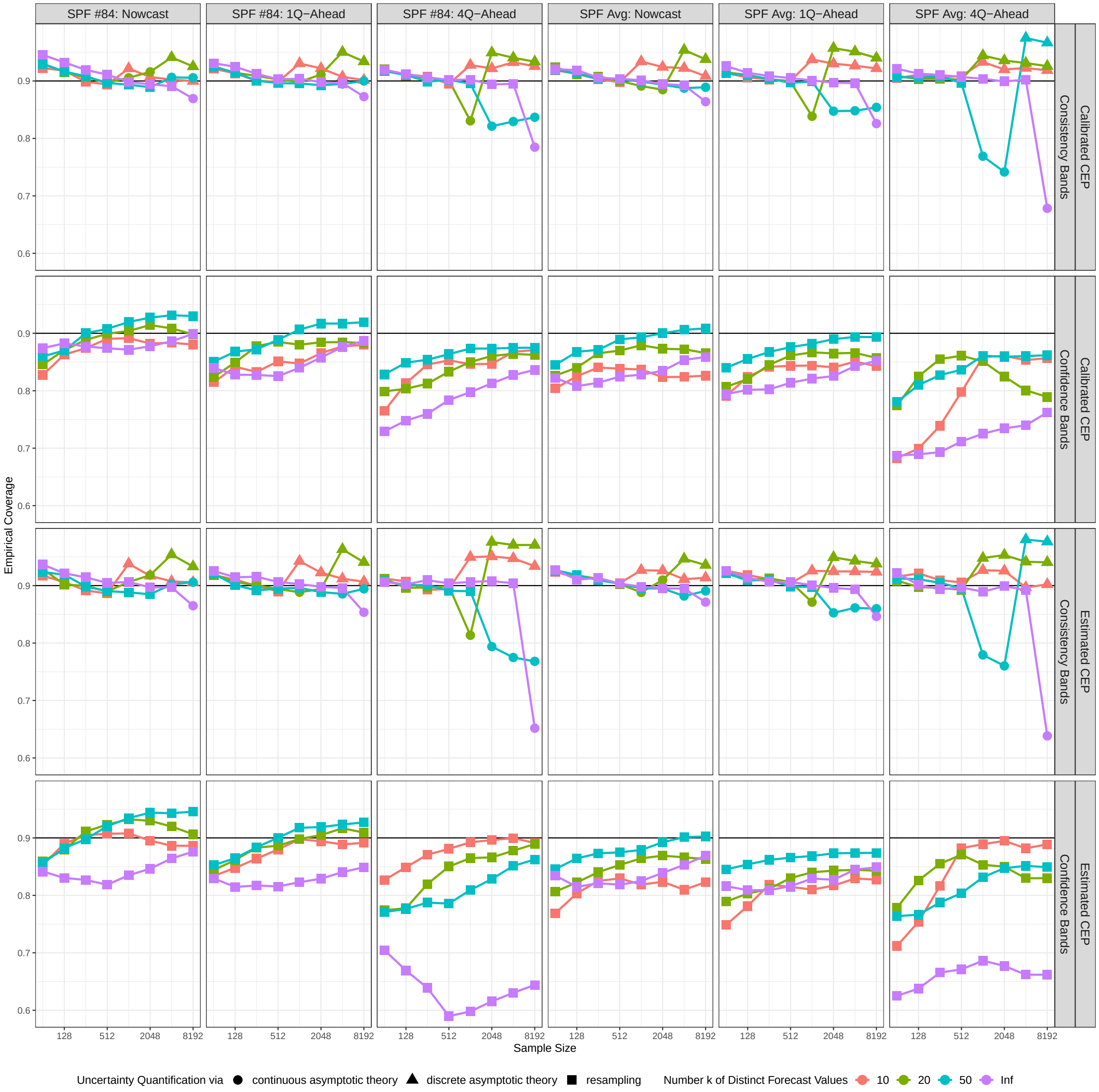

Fig. S9. Coverage rates in the setting of Fig. S6, with parameter values informed by the SPF U.S. GDP recessions data described in Table S1.

B. Statistical efficiency of CORP: Data-driven simulations. Next we extend the simulation study on the statistical efficiency of the CORP approach, as described in Appendix B of the main paper, to the data-driven scenarios introduced in Section A.

Specifically, Fig. 6 in the main article and Figs. S10–S13 in this supplementary document compare the efficiency of the CORP approach to estimating CEPs to that of estimates under the binning and counting approach, using either a fixed number of $m = 5$, 10, or 50 bins, or a flexible number of $m(n) = [n^\alpha]$ quantile-based bins, where $[x]$ denotes the smallest integer less than or equal to $x \in \mathbb{R}$, n is the sample size, and $\alpha = \frac{1}{6}$, $\frac{1}{3}$, or $\frac{1}{2}$. With reference to Section S2, the quantile-based approach uses variant Q. Throughout, we plot the (log) mean squared error (MSE) of the respective estimate against the (log) sample size n. The underlying continuous and discrete simulation settings in Figs. S10–S13 are driven by the data sets in Table S1, as described in Subsection A, except that we discretize to $k = 10$ and $k = 50$ unique forecast values only.

We see that under nearly all scenarios, and for all sample sizes considered, the CORP method exhibits the highest efficiency. The only exceptions are in Fig. S13 within the columns that correspond to probability forecasts of U.S. GDP recessions at a prediction horizon of four quarters ahead — the very same cases that we had flagged in the previous section. Here, CORP estimates fail to outperform estimates under the binning and counting approach that use very small numbers of bins only. The underlying reason is that the true CEP is nearly horizontal in these cases, as quantified by the very low values of α_C and β_C in Table S1, and reflecting the drastic lack of discrimination ability in these forecasts, visualized in the respective CORP reliability diagrams in Fig. S1(d). If the true CEP function is genuinely horizontal — a situation that is hardly ever seen in pratice, and counter to the assumptions of the large sample results in Appendix B of the main paper — the highest estimation efficiency is achieved by employing the trivial method of using a single bin only, spanning the entire unit interval. In these very specific simulation examples, the assumed true CEP function is nearly horizontal, and the finite sample MSE is slightly lower under binning and counting than under the CORP approach, provided the number of bins used remains small (e.g., 5 fixed or $n^{1/6}$ bins). However, it is exactly these bin choices that perform particularly poorly in all other settings, where the assumed true CEP curves are meaningfully increasing, as in the vast majority of applications. Furthermore, even in the exceptional cases the CORP approach retains the benefit of superior interpretability, as illustrated in Fig. S5.

S5. Properties of score decompositions

In this section we supplement Appendix C in the main paper by providing proofs of the theoretical results, and by constructing examples where the respective claims fail under (re)calibration techniques other than isotonic regression.

A. Proofs. For convenience, we recall the statements of Theorem 1 and Theorem 2 in Appendix C of the main article.

Theorem 1 Given any set of original forecast values and associated binary events, suppose that we apply the PAV algorithm to generate a (re)calibrated forecast, and use the marginal event frequency as reference forecast. Then, for every proper scoring rule S, the score decomposition defined by Eqs. [2] and [3] in the main article satisfies the following:

(a) $\mathsf{MCB} = \bar{\mathsf{S}}_\mathsf{X} - \bar{\mathsf{S}}_\mathsf{C} \geq 0$ with equality if the original forecast itself is calibrated.

(b) $\mathsf{MCB} > 0$ if the score is strictly proper and the original forecast is not calibrated.

(c) $\mathsf{DSC} = \bar{\mathsf{S}}_\mathsf{R} - \bar{\mathsf{S}}_\mathsf{C} \geq 0$ with equality if the (re)calibrated forecast is constant.

(d) $\mathsf{DSC} > 0$ if the score is strictly proper and the (re)calibrated forecast is not constant.

(e) The decomposition is exact.

Proof of Theorem 1 The claims in (a) and (c) rely on the fact that the PAV algorithm generates a (re)calibrated forecast that is no worse than the original forecast in terms of any proper scoring rule (Barlow et al. (27), Thm. 1.10; Fawcett (28); Brümmer and Du Preetz (29)). If the original forecast itself is calibrated, then the associated CEP curve is isotonic and the PAV algorithm leaves it unchanged. If the PAV algorithm generates a constant forecast, the constant equals the marginal event frequency $\bar{y}$ and, therefore, the PAV (re)calibrated and the reference forecast values are the same.

The statements in (b) and (d) follow from the equivalence of (i) and (iii) in Theorem 2.11 of Gneiting and Ranjan (30) in concert with Theorem 3 of Holzmann and Eulert (31). Finally, the claim in (e) is immediate from the definition of the decomposition. □

Theorem 2 Under the Brier score, if the sequence $o_1/n_1, \ldots, o_k/n_k$ is nondecreasing, then $\mathsf{MCB} = \mathsf{REL}$ and $\mathsf{DSC} = \mathsf{RES}$, respectively.

Proof As the sequence $o_1/n_1, \ldots, o_k/n_k$ is nondecreasing, the PAV-calibrated probabilities $\hat{z}_j$ satisfy $\hat{z}_j = o_j/n_j$, for $j = 1, \ldots, k$. Adopting arguments in Dawid (32) or in the Appendix of Siegert (33), we see that $\mathsf{MCB} = \bar{\mathsf{S}}_\mathsf{X} - \bar{\mathsf{S}}_\mathsf{C} = \mathsf{REL}$ and $\mathsf{DSC} = \mathsf{RES}$, respectively. □

B. Counterexamples under (re)calibration techniques other than isotonic regression via the PAV algorithm. Claims (a), (b), (c), and (d) in Theorem 1 depend critically on the assumption that the PAV-transformed probabilities serve as the (re)calibrated forecast, and the marginal event frequency as the reference forecast. Only part (e) holds in general, regardless of the specific choices for the (re)calibrated forecast and the reference forecast.

In the following, we give specific examples in order to show that under alternatives choices of (re)calibration techniques, such as logistic regression (33) or Beta CDF fits (1), the claims in parts (a), (b), (c), and (d) of Theorem 1 may fail.

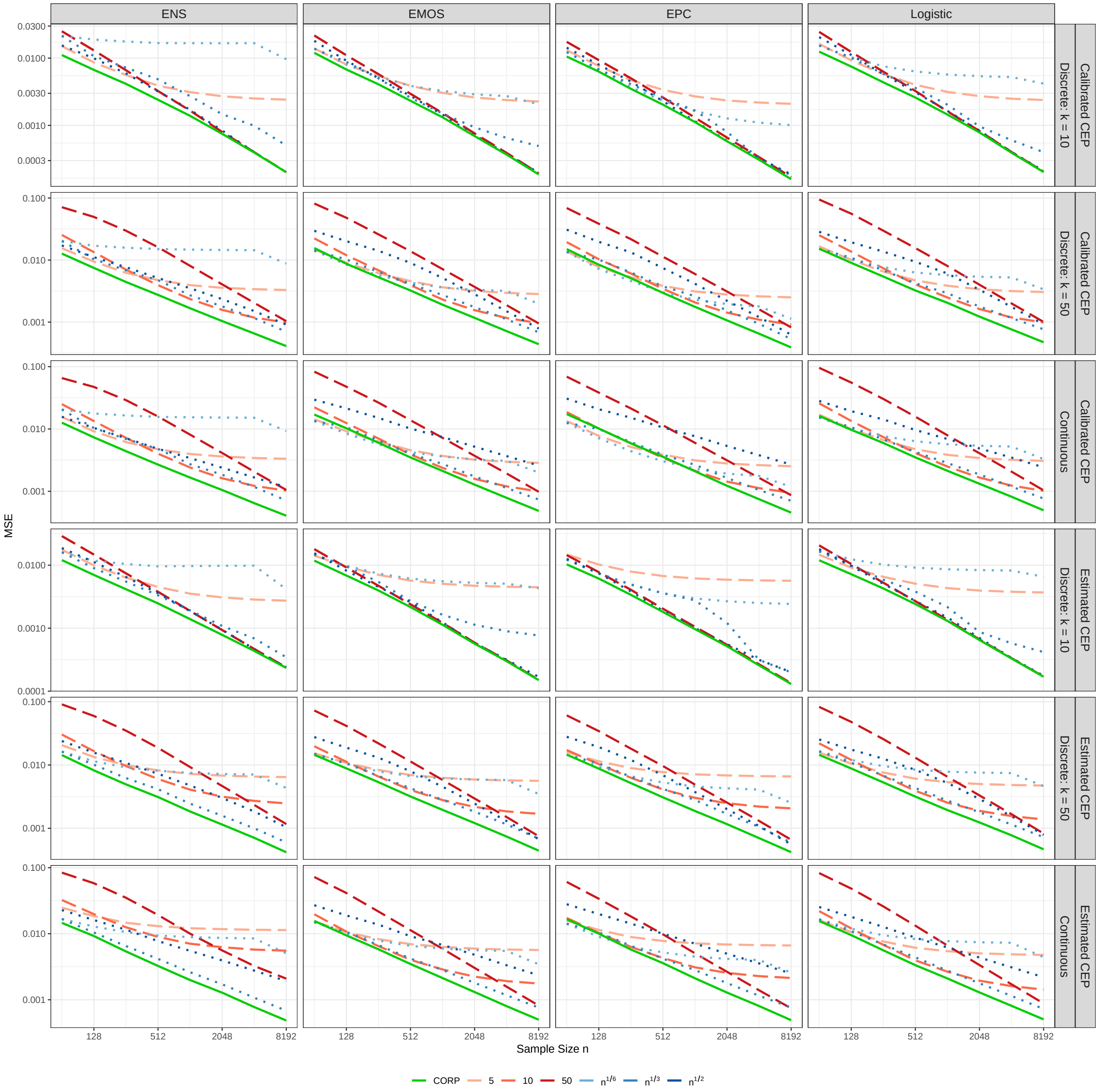

Fig. S10. Mean squared error (MSE) of CEP estimates in CORP reliability diagrams for samples of size n, in comparison to the binning and counting approach with $m = 5$, 10, or 50 fixed bins, or $m(n) = \lceil n^\alpha \rceil$ quantile-based bins, where $\alpha = \frac{1}{6}$, $\frac{1}{3}$, or $\frac{1}{2}$. Note the log-log scale. The columns correspond to Beta distributions for the forecast values with parameter values α_P and β_P associated with probability of precipitation forecasts at Niamey, Niger, as listed in Table S1. The upper three rows show results in the case of a calibrated CEP, when the binary outcomes are Bernoulli draws from the forecast probabilities. The bottom three rows show results under CEP functions that equal Beta CDFs with parameter values α_C and β_C as also listed in Table S1. For further details, see Appendix A in the main article and Parts A and B of Section S4.

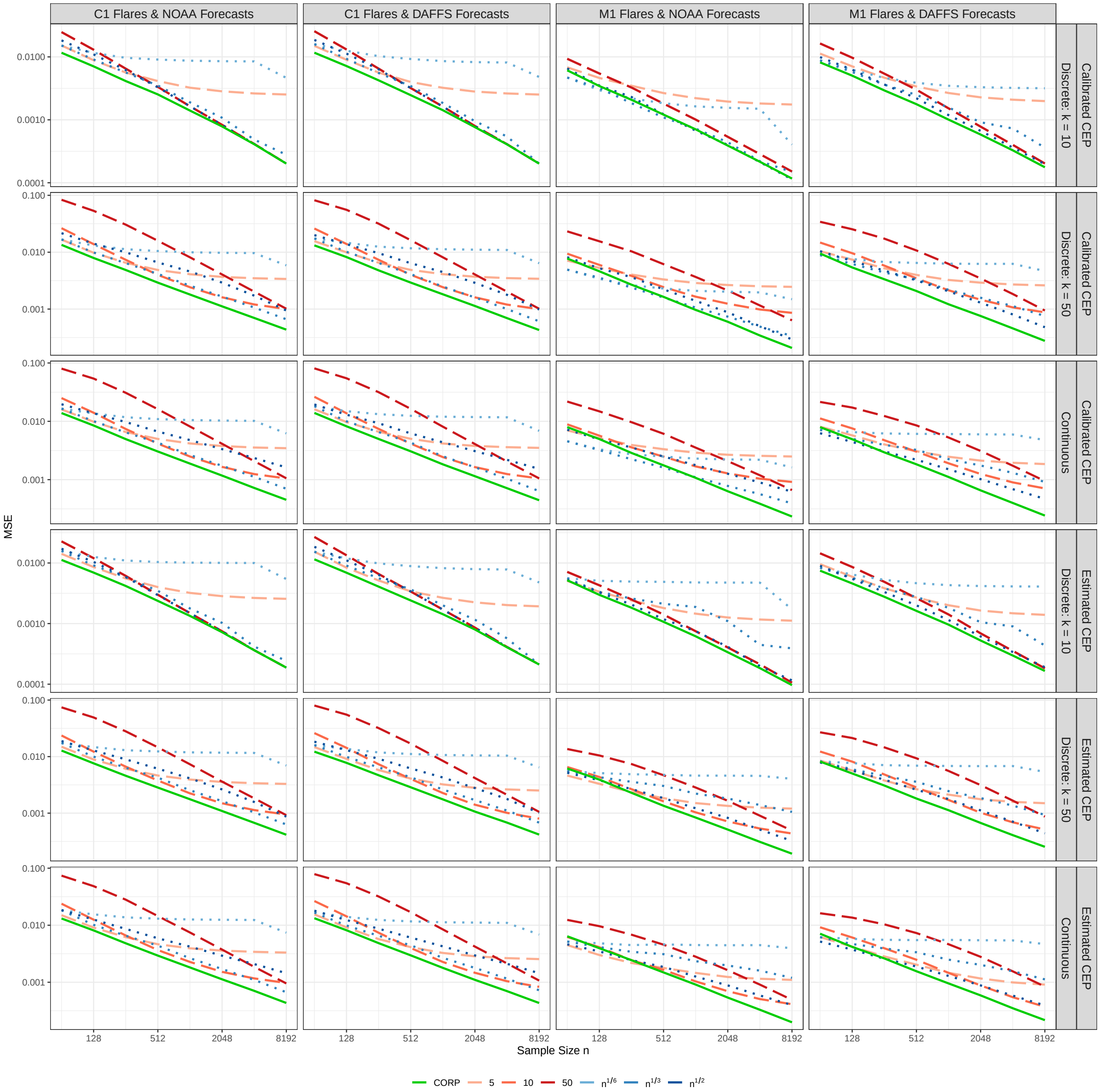

Fig. S11. Statistical efficiency of CEP estimates in the setting of Fig. S10, now driven by the solar flares data described in Table S1.

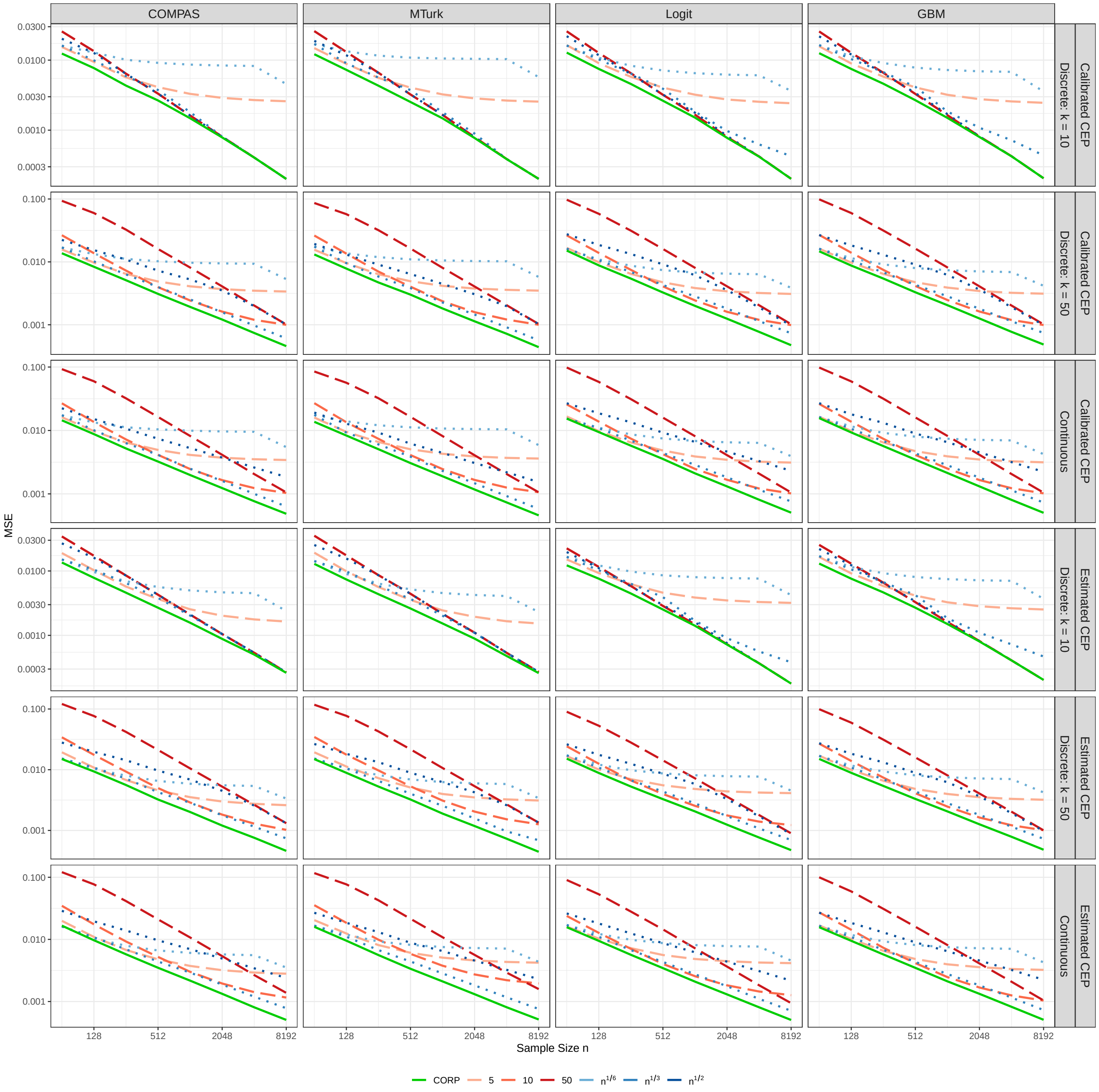

Fig. S12. Statistical efficiency of CEP estimates in the setting of Fig. S10, now driven by the recidivism data described in Table S1.

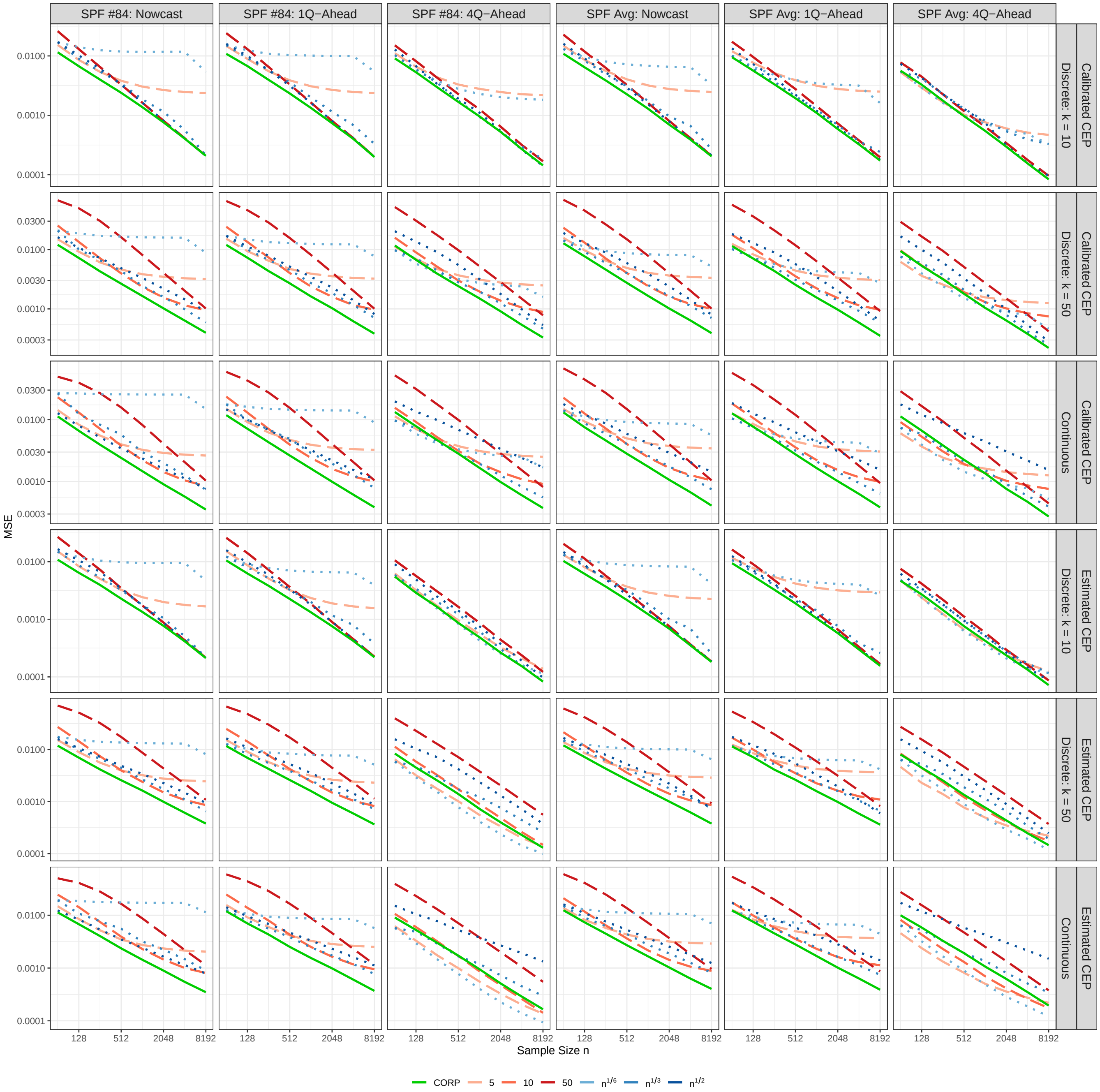

Fig. S13. Statistical efficiency of CEP estimates in the setting of Fig. S10, now driven by the SPF U.S. GDP recession data described in Table S1.

(a) Consider the forecast–realization pairs $(x_1, y_1) = (.02, 0), (x_2, y_2) = (.48, 1), (x_3, y_3) = (.52, 0)$, and $(x_4, y_4) = (.98, 1)$, and suppose that the (re)calibrated forecast is based on logistic regression, estimated by maximum likelihood. Then, using the Brier score yields $\bar{\mathsf{S}}_\mathsf{X} = .135$ for the original forecast and $\bar{\mathsf{S}}_\mathsf{C} = .146$ for the calibrated forecast, whence $\mathsf{MCB} = \bar{\mathsf{S}}_\mathsf{X} - \bar{\mathsf{S}}_\mathsf{C} < 0$. An intuitive explanation is that logistic regression enforces a smooth fit with a single inflection point only, whereas in this example a continuously differentiable recalibration function would need to have at least three inflection points in order to move .02 towards 0, .48 and .52 towards 1/2, and .98 towards 1. To see this, note that the average slopes of the recalibration function in each of the intervals $(0, .02)$, $(.48, .52)$, and $(.98, 1)$ would need to be less than one, whereas the average slopes in the intervals $(.02, .48)$ and $(.52, .98)$ would need to be larger than one. Finally, as the identity function on the unit interval does not lie in the model space of logistic regression, it is possible to deteriorate calibration compared to the original forecast. Of course, one might argue that logistic regression is a poor method for recalibrating probability forecasts, but this is exactly our point: Instead, isotonic regression implemented via the PAV algorithm ought to be used.

(b) The example from part (a) applies, as $\mathsf{MCB} < 0$ even though the Brier score is strictly proper and the original forecast fails to be calibrated.

(c) Unlike in the case of the MCB component, we expect negative DSC components to hardly ever occur in practice, regardless of the (re)calibration method used. Nevertheless, toy examples of this type can be constructed. For instance, the use of Beta CDFs has been proposed for (re)calibration. For the forecast–realization pairs $(x_1, y_1) = (.20, 1)$ and $(x_2, y_2) = (.70, 1)$ we have $\bar{y} = 1$, and under the Brier score $\bar{\mathsf{S}}_\mathsf{R} = 0$ while $\bar{\mathsf{S}}_\mathsf{C} > 0$ under (re)calibration, given that Beta CDFs are strictly increasing on the unit interval, and so $\mathsf{DSC} = \bar{\mathsf{S}}_\mathsf{R} - \bar{\mathsf{S}}_\mathsf{C} < 0$.

(d) Consider the same setting as in (c), but with the forecast–realization pairs $(x_1, y_1) = (.00, 0), (x_2, y_2) = (.00, 0), (x_3, y_3) = (.00, 1), (x_4, y_4) = (1.00, 0)$, and $(x_5, y_5) = (1.00, 1)$. The Brier score is strictly proper, $\bar{y} = \frac{2}{5}$, and the (re)calibrated forecast is nonconstant. Then $\bar{\mathsf{S}}_\mathsf{R} = .24$ and $\bar{\mathsf{S}}_\mathsf{C} = \bar{\mathsf{S}}_\mathsf{X} = .40$, as Beta CDFs leave values of zero or one unchanged, and consequently $\mathsf{DSC} = \bar{\mathsf{S}}_\mathsf{R} - \bar{\mathsf{S}}_\mathsf{C} < 0$.

S6. CORP discrimination diagrams

Reliability diagrams serve as purposefully designed, visual diagnostic tools that allow researchers and practitioners to detect and diagnose miscalibration. Alternatively, forecast producers and forecast users might have a primary interest in the effects of recalibration, as performed by the PAV algorithm, with the effect of altering the marginal distribution of the forecast values. In such cases, a variant of the CORP reliability diagram, suggested to us by a referee, and hereinafter referred to as CORP *discrimination diagram*, is a powerful tool. CORP discrimination diagrams feature the graph of the CORP CEP estimate, just like a CORP reliability diagram does, and furthermore histograms of both the original and the PAV-(re)calibrated forecast values, which we place at top and at right, respectively. In order to resolve the change implied by the PAV (re)calibration, we use 100 equidistant bins in these histograms. Furthermore, we show the diagonal, which indicates calibration, just as in a reliability diagram, and we indicate the unconditional event probability in both horizontal and vertical line segments. In the framework of CORP, the constant forecast that attains this value serves as reference forecast. Finally, CORP discrimination diagrams show the values of the MCB, DSC, and UNC components of the CORP Brier score decomposition; cf. the section on score decompositions and Appendix C in the main article and Section S5 in this supplement.

Figure S14 shows CORP discrimination diagrams for the data sets in Table S1, and the displays can be compared to the default CORP reliability diagrams in Figs. 1(b,d,f) and 2(d) in the main article and Fig. S1 in this supplement. In a nutshell, CORP reliability diagrams put the focus on calibration; whereas CORP discrimination diagrams address both calibration and discrimination, a benefit that comes at the expense of a less focused and perhaps more crowded display. In our software implementation in R (15, 34) both CORP reliability diagrams and CORP discrimination diagrams can be generated by the `plot()` or `autoplot()` commands, where the `type` argument determines the kind of diagram.

References

1. Ranjan R, Gneiting T (2010) Combining probability forecasts. *Journal of the Royal Statistical Society Series B: Methodological* 72:71–91.
2. Dimitriadis T, Jordan AI (2020) Replication material. Available at https://github.com/TimoDimi/replication_DGJ20.
3. Vogel P, et al. (2021) Statistical forecasts for the occurrence of precipitation outperform global models over northern tropical africa. *Geophysical Research Letters.* 48, e2020GL091022.
4. Leka KD, et al. (2019) A comparison of flare forecasting methods. II. Benchmarks, metrics, and performance results for operational solar flare forecasting systems. *The Astrophysical Journal Supplement Series* 243(2):36.
5. Crown MD (2012) Validation of the NOAA Space Weather Prediction Center's solar flare forecasting look-up table and forecaster-issued probabilities. *Space Weather* 10:S06006.
6. Leka KD, Barnes G, Wagner E (2018) The NWRA classification infrastructure: Description and extension to the Discriminant Analysis Flare Forecasting System (DAFFS). *Journal of Space Weather and Space Climate* 8:A25.
7. Dressel J, Farid H (2018) The accuracy, fairness, and limits of predicting recidivism. *Science Advances* 4:eaao5580.
8. Bansak K (2019) Can nonexperts really emulate statistical learning methods? A comment on "The accuracy, fairness, and limits of predicting recidivism". *Political Analysis* 27:370–380.

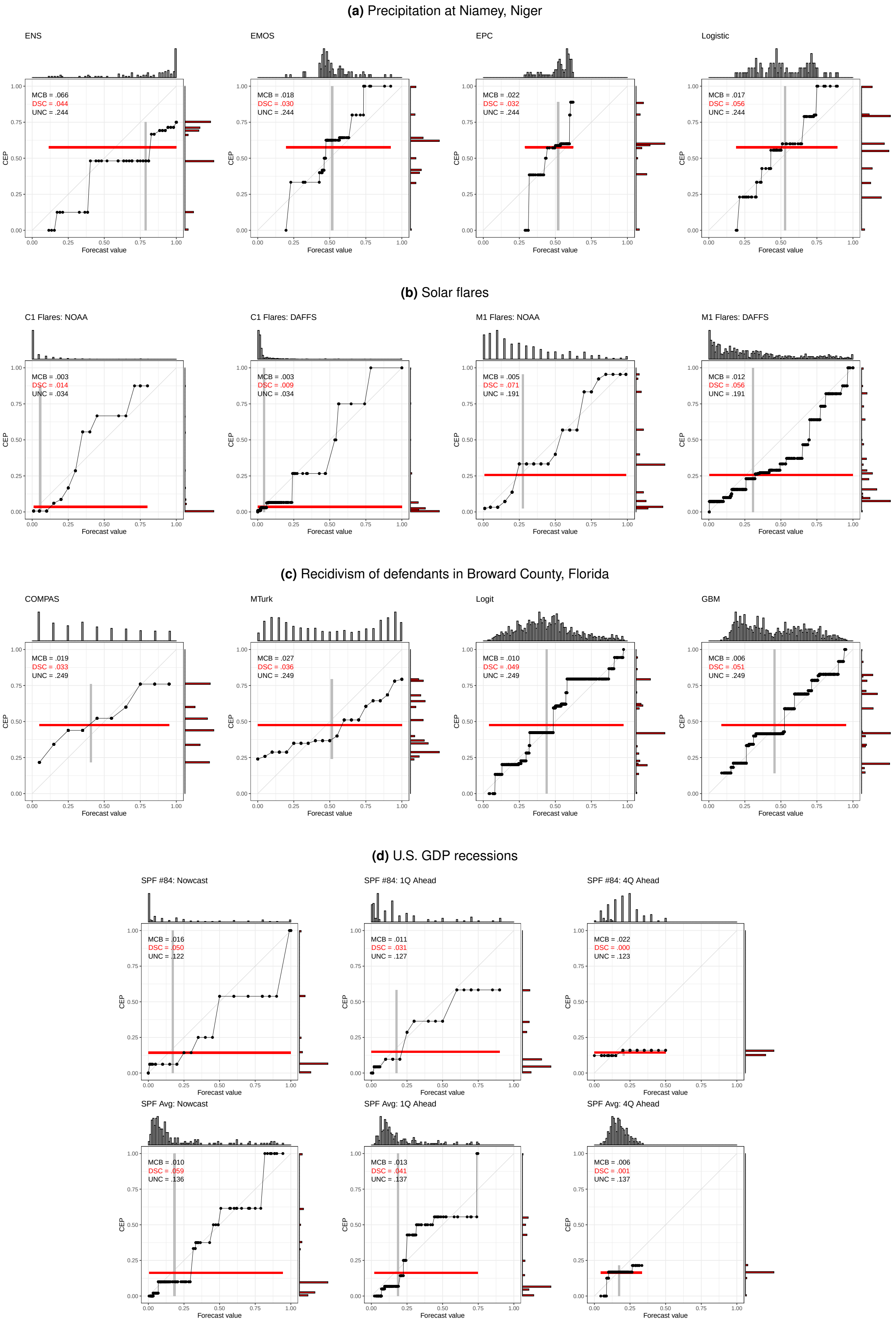

Fig. S14. CORP discrimination diagrams for the probability forecasts in the four data sets described in Table S1. See Section S6 for details and Fig. S1 for the comparison to default CORP reliability diagrams.

9. Croushore D (1993) Introducing: The Survey of Professional Forecasters. Federal Reserve Bank of Philadelphia Business Review Nov./Dec. 1993, available at http://scholarship.richmond.edu/cgi/viewcontent.cgi?article=1012&context=economics-faculty-publications.
10. Croushore D, Stark T (2001) A real-time data set for macroeconomists. *Journal of Econometrics* 105:111–130.
11. Lahiri K, Wang JG (2013) Evaluating probability forecasts for GDP declines using alternative methodologies. *International Journal of Forecasting* 29:175–190.
12. Barnes G, et al. (2016) A comparison of flare forecasting methods. I. Results from the "all-clear" workshop. *The Astrophysical Journal* 829(2):89.
13. Leka KD, Park SH (2019) A comparison of flare forecasting methods II: Data and supporting code. Available at https://doi.org/10.7910/DVN/HYP74O.
14. Angwin J, Larson J, Mattu S, Kirchner L (2016) Machine bias: There's software used across the country to predict future criminals. And it's biased against blacks. Available at https://www.propublica.org/article/machine-bias-risk-assessments-in-criminal-sentencing.
15. R Core Team (2020) *R: A Language and Environment for Statistical Computing.* R Foundation for Statistical Computing, Vienna, Austria. Available at https://www.R-project.org/.
16. Stephenson DB, Coelho CAS, Jolliffe IT (2008) Two extra components in the Brier score decomposition. *Weather and Forecasting* 23:752–757.
17. Hosmer DW, Lemeshow S (1980) Goodness of fit tests for the multiple logistic regression model. *Communications in Statistics — Theory and Methods* 9:1043–1069.
18. Bertolini G, D'Amico R, Nardi D, Tinazzi A, Apolone G (2000) One model, several results: The paradox of the Hosmer–Lemeshow goodness-of-fit test for the logistic regression model. *Journal of Epidemiology and Biostatistics* 5:251–253.
19. Kuss O (2002) Global goodness-of-fit tests in logistic regression with sparse data. *Statistics in Medicine* 21(24):3789–3801.
20. Allison PD (2014) Measures of fit for logistic regression. Paper 1485–2014, SAS Global Forum, Washington DC.
21. Tutz G (2011) *Regression for Categorical Data.* (Cambridge University Press, Cambridge).
22. Harrell Jr FE (2015) *Regression Modeling Strategies: With Applications to Linear Models, Logistic Regression, and Survival Analysis.* (Springer, Cham, Switzerland), second edition.
23. Agresti A (2013) *Categorical Data Analysis.* (Wiley, Hoboken, New Jersey), third edition.
24. El Barmi H, Mukerjee H (2005) Inferences under a stochastic ordering constraint. *Journal of the American Statistical Association* 100:252–261.
25. Groeneboom P, Wellner JA (2001) Computing Chernoff's distribution. *Journal of Computational and Graphical Statistics* 10:388–400.
26. Wright FT (1981) The asymptotic behavior of monotone regression estimates. *Annals of Statistics* 9:443–448.
27. Barlow RE, Bartholomew DJ, Bremner JM, Brunk HD (1972) *Statistical Inference Under Order Restrictions: The Theory and Application of Isotonic Regression.* (Wiley, New York).
28. Fawcett T, Niculescu-Mizil A (2007) PAV and the ROC convex hull. *Machine Learning* 68:97–106.
29. Brümmer N, Du Preez J (2013) The PAV algorithm optimizes binary proper scoring rules. Preprint, available at https://arxiv.org/abs/1304.2331.
30. Gneiting T, Ranjan R (2013) Combining predictive distributions. *Electronic Journal of Statistics* 7:1747–1782.
31. Holzmann H, Eulert M (2014) The role of the information set for forecasting — with applications to risk management. *Annals of Applied Statistics* 8:595–621.
32. Dawid AP (1986) Probability forecasting, in *Encyclopedia of Statistical Sciences.* (Wiley-Interscience) Vol. 7, pp. 210–218.
33. Siegert S (2017) Simplifying and generalising Murphy's Brier score decomposition. *Quarterly Journal of the Royal Meteorological Society* 143:1178–1183.
34. Dimitriadis T, Jordan AI (2020) reliabilitydiag: Reliability diagrams using isotonic regression. R package version 0.1.3, available at https://cran.r-project.org/package=reliabilitydiag.
